# Supplementary material for: Client proximity enhancement inside cellular membrane-less compartments governed by client-compartment interactions
Source: Nat Commun. 2020 Nov 6;11:5642. doi: 10.1038/s41467-020-19476-4 (PMC7648067; doi:10.1038/s41467-020-19476-4)
Supplement: Supplementary file 1 — Supplementary Information [file 41467_2020_19476_MOESM1_ESM.pdf]

Supplementary Information

Client proximity enhancement inside cellular membrane-less compartments governed by client-compartment interactions

Daesun Song[1], Yongsang Jo[1], Jeong-Mo Choi[2,3], and Yongwon Jung[1]*

[1]*Department of Chemistry, Korea Advanced Institute of Science and Technology, Daejeon 34141, Republic of Korea. E-mail: ywjung@kaist.ac.kr*

[2]*Natural Science Research Institute, Korea Advanced Institute of Science and Technology, Daejeon 34141, Republic of Korea.*

[3]*Department of Chemistry, Pusan National University, Busan 46241, Republic of Korea*

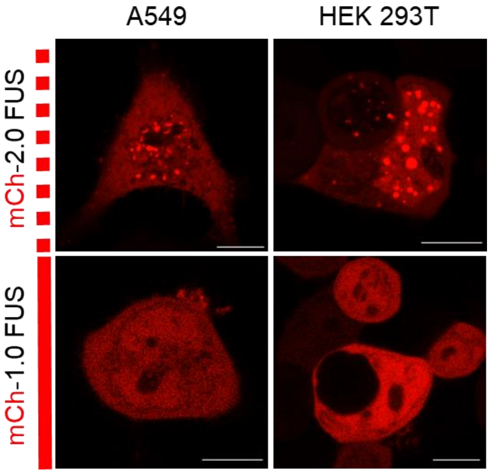

Supplementary Figure 1 Cellular protein condensate formation of mCherry(mCh)-fused 2.0 FUS (top) and 1.0 FUS (bottom) in A549 and HEK 293T cells. Scale bars: 10 μm.

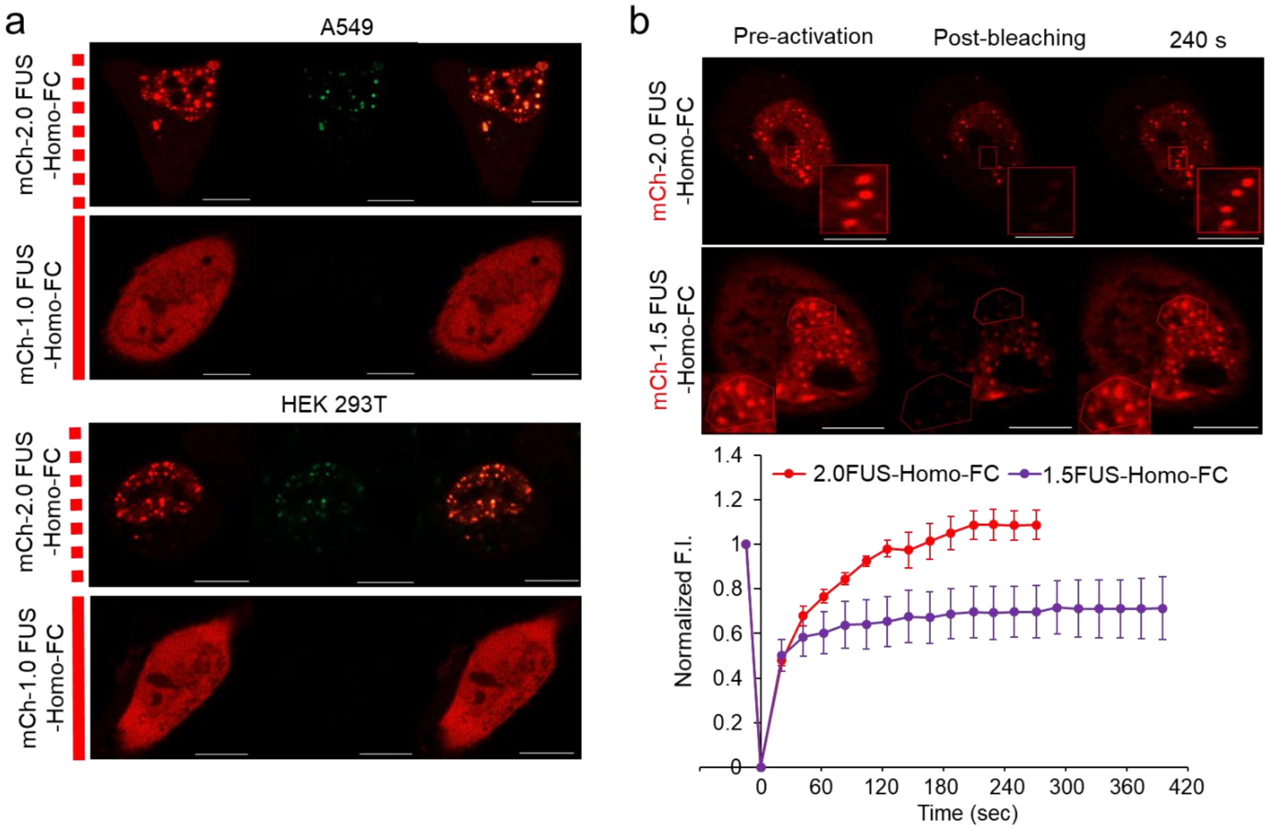

Supplementary Figure 2 Fluorescence complementation of scaffold-fused probes inside repeated-FUS compartments. **(a)** Fluorescence (mCh and GFP) images of cells (A549 and HEK 293T cells) expressing mCh-2.0 FUS-Homo-FC or mCh-1.0 FUS-Homo-FC. **(b)** FRAP recovery images and profiles of scaffold proteins of mCh-2.0 FUS-Homo-FC and mCh-1.5 FUS-Homo-FC condensates. Scale bars: 10 μm. Data (point) are presented as mean values with +/- 1 s.d. as error bars (n = 33 biologically independent cells examined over three independent experiments).

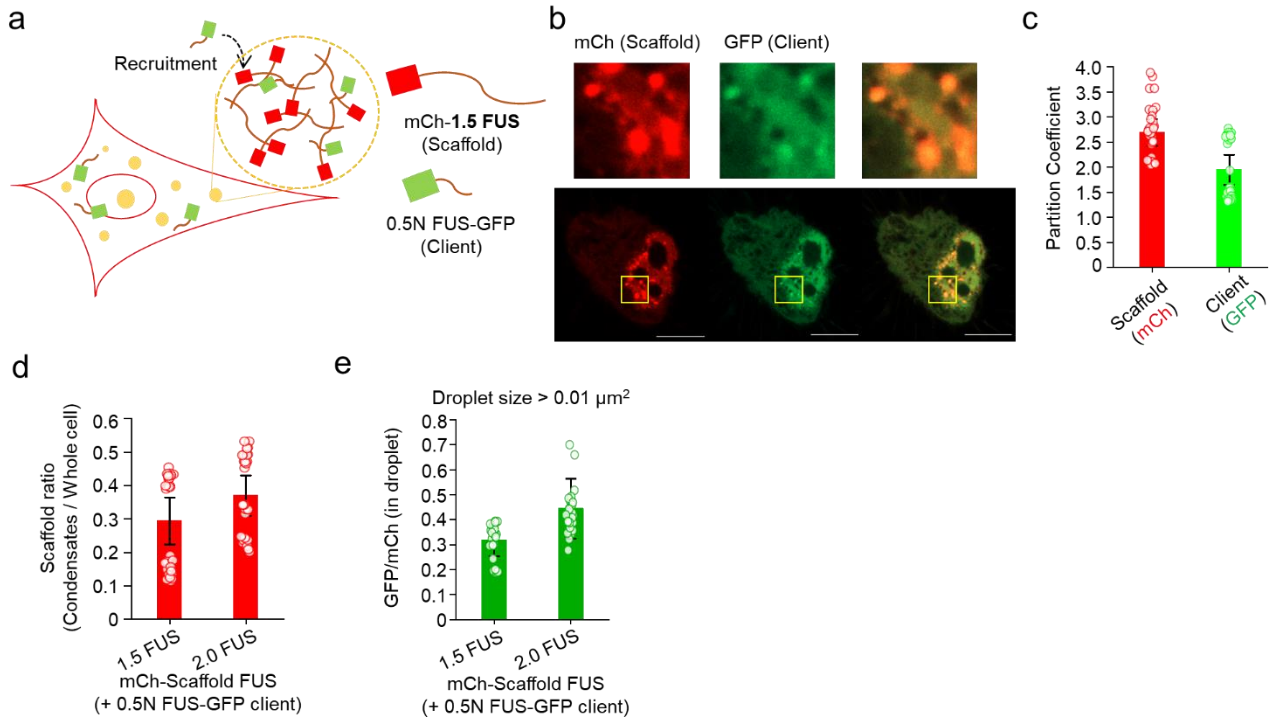

Supplementary Figure 3 Fluorescence complementation of recruited client probes inside repeated-IDP compartments. **(a)** Schematic illustration of cellular condensate formation of mCh-1.5 FUS and 0.5N FUS-GFP recruitment. **(b)** Fluorescence (mCh and GFP) images of cells expressing mCh-1.5 FUS (scaffold) and 0.5N FUS-GFP (client). Scale bars: 10 μm. **(c)** Partition coefficient of scaffold (mCh) and client (GFP) inside/outside condensates. Data (bar) are presented as mean values with +/- 1 s.d. as error bars (n = 33 for Scaffold, n = 31 for Client; n: cells examined over three independent experiments). **(d)** 1.5 FUS- or 2.0 FUS-fused total mCh (scaffold) signal ratios between condensates and whole cells with co-expressed 0.5N FUS-GFP. Data are presented as mean values with +/- 1 s.d. as error bars (n = 33 cells from three independent experiments). **(e)** Total GFP (0.5N FUS-GFP) and mCh ratios inside droplets for 1.5 FUS or 2.0 FUS condensates. Data are presented as mean values with +/- 1 s.d. as error bars (n = 33 cells from three independent experiments).

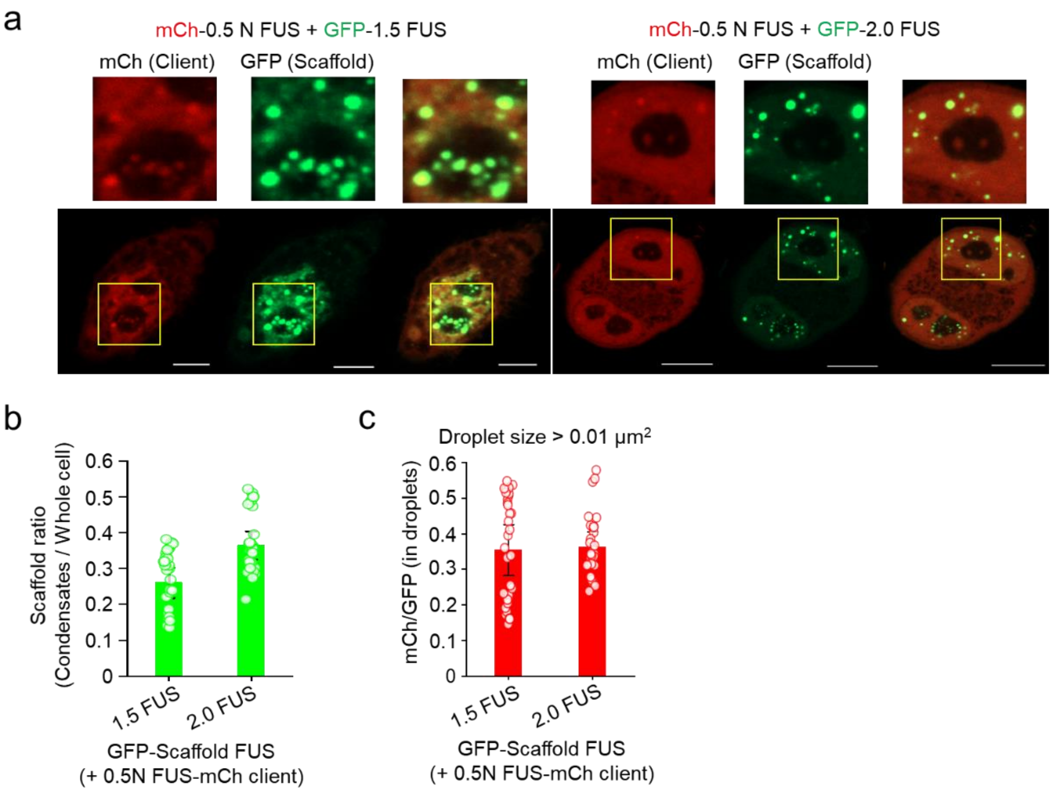

Supplementary Figure 4 Client (mCh-0.5N FUS) recruitment into GFP-FUS condensates. **(a)** Fluorescence (mCh and GFP) images of cells expressing GFP-1.5 FUS (left) or GFP-2.0 FUS (right) (scaffold) and mCh-0.5N FUS (client). Scale bars: 10 μm. **(b)** 1.5 FUS- or 2.0 FUS-fused total GFP (scaffold) signal ratios between condensates and whole cells with co-expressed 0.5N FUS-mCh. Data are presented as mean values with +/- 1 s.d. as error bars (n = 33 cells from three independent experiments). **(c)** Total mCh (0.5N FUS-mCh) and GFP ratios inside droplets for 1.5 FUS- or 2.0 FUS-GFP condensates. Data are presented as mean values with +/- 1 s.d. as error bars (n = 33 cells from three independent experiments).

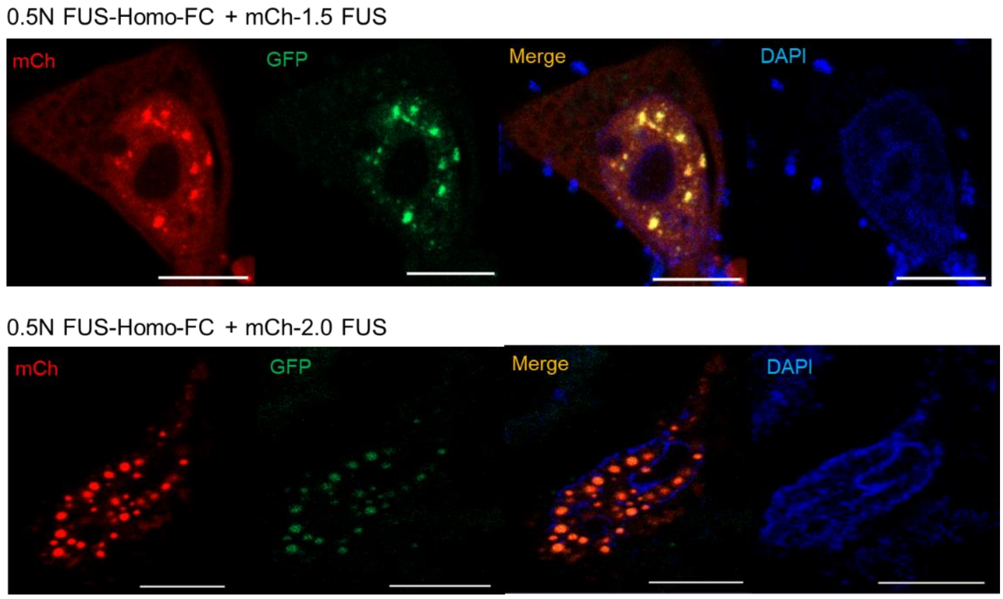

Supplementary Figure 5 Fluorescence (mCh and GFP) images of cells expressing mCh-1.5 FUS or mCh-2.0 FUS (scaffold) and 0.5N FUS-Homo-FC (client) with DAPI images. Experimental conditions were same as Fig. 3f, but cells were fixed and stained with DAPI. Scale bars: 10 μm.

Note: In the Fig. 3f experiments, live cells were imaged without DAPI staining to simultaneously conduct FRAP. In addition, as discussed in the text, FUS condensates formed mostly in the nucleus. Since images were obtained with a focus in the middle of cells (focusing around the nucleus), the size of the nucleus was rather large.

a

Without condensates

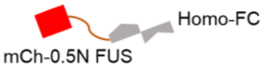

mCh-0.5N FUS Homo-FC			
Cell number	mCh expression signal	Complementation signal (GFP)	GFP/mCh
1	4045.482	79.208	0.020
2	4269.757	83.214	0.019
3	4472.902	83.109	0.019
4	4886.731	79.882	0.016
5	5184.573	115.671	0.022
6	6255.844	142.184	0.023
7	6936.202	157.895	0.023
8	7590.848	157.597	0.021
9	8133.960	177.250	0.022
10	8349.015	189.552	0.023
11	9192.046	157.328	0.017
12	9326.019	203.848	0.022
13	9618.175	184.410	0.019
14	11746.817	235.791	0.020
15	11840.405	226.471	0.019
16	13222.453	290.485	0.022
17	13715.289	300.533	0.022
18	13954.789	314.684	0.023
19	14361.498	294.366	0.020
20	14812.316	307.585	0.021
21	15101.871	267.068	0.018
22	15267.875	331.691	0.022
23	15436.357	297.484	0.019
24	16052.626	346.424	0.022
25	16333.389	375.550	0.023
26	16686.922	359.348	0.022
27	17557.232	396.608	0.023
28	17919.253	427.651	0.024
29	17978.603	412.730	0.023
30	18070.161	367.696	0.020
31	18995.214	397.490	0.021
32	19100.647	443.818	0.023
33	19702.598	456.331	0.023
AVE			0.021
STDEV			0.002

b

With condensates

Scaffold		Client	
	mCh-2.0 FUS	0.5N FUS Homo-FC	
Cell number	mCh expression signal	Complementation signal (GFP)	GFP/mCh
1	4016.04	2907.90	0.72
2	4394.21	2508.31	0.57
3	4814.04	3184.74	0.66
4	4934.44	4702.67	0.95
5	5457.53	2141.92	0.39
6	5641.48	4045.30	0.72
7	6190.37	2994.92	0.48
8	6349.30	5258.70	0.83
9	7450.23	7263.99	0.98
10	8265.09	6840.74	0.83
11	9226.12	3806.29	0.41
12	10579.62	5758.65	0.54
13	10995.02	8210.74	0.75
14	11065.82	9286.94	0.84
15	11625.30	4578.44	0.39
16	12426.31	10979.88	0.88
17	13293.90	5023.93	0.38
18	14818.74	9083.05	0.61
19	15415.55	14861.76	0.96
20	15850.06	9113.33	0.57
21	16050.16	15573.91	0.97
22	16189.92	14039.31	0.87
23	16572.13	14051.72	0.85
24	16887.36	10170.15	0.60
25	17197.93	16599.60	0.97
26	17657.12	15578.96	0.88
27	18166.45	12794.58	0.70
28	18388.00	13778.14	0.75
29	18446.26	15508.63	0.84
30	19138.39	15187.58	0.79
31	19442.63	8039.69	0.41
32	19671.12	14685.98	0.75
33	19911.39	14290.86	0.72
AVE			0.71
STDEV			0.19

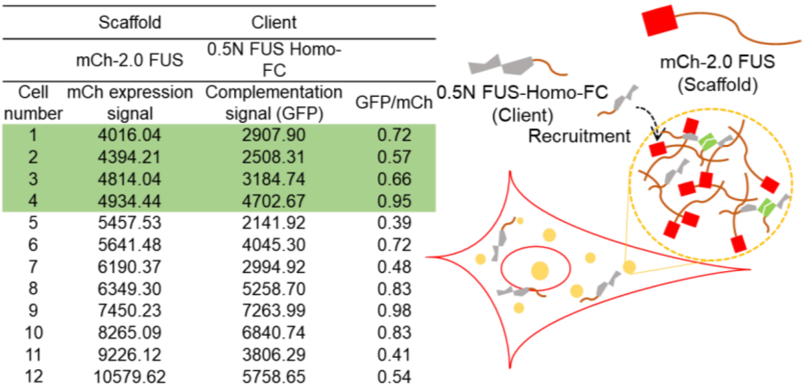

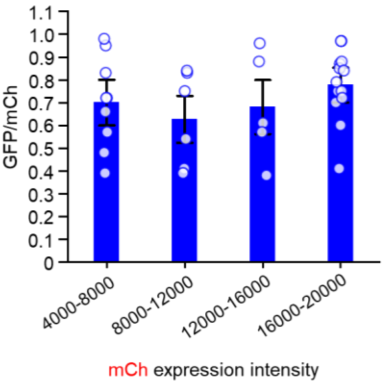

Supplementary Figure 6 Complementation signals at various protein expression levels. **(a)** Protein expression signals (mCh) and FC signals of mCh-0.5N FUS-Homo-FC without condensates for examined 33 cells. Four cells with the highest protein expression (mCh) are indicated (gray). **(b)** Scaffold (mCh-2.0 FUS) expression signals and 0.5N FUS-Homo-FC complementation signals of examined 33 cells. Four cells with the lowest expression (mCh) are indicated (green). Cellular GFP and mCh signal ratios at various protein expression levels are shown in the right graph. Expression levels are categorized based on mCh expression intensities. Data are presented as mean values with +/- 1 s.d. as error bars (n = 9 cells for 4000-8000, n = 6 cells for 8000-12000, n = 5 cells for 12000-16000, n = 13 cells for 16000-20000; from three independent experiments).

Note I: mCh-0.5N FUS-Homo-FC alone without condensates did not show any complementation signals in all examined cells regardless of 5-fold expression level variation (Supplementary Fig. 6a). However, 0.5N FUS-Homo-FC with FUS condensates showed consistently high complementation GFP/mCh values even with low mCh-2.0 FUS scaffold (therefore low 0.5N FUS-Homo-FC client)

levels in cells.

Note II: In addition, FC signals of mCh-0.5N FUS-Homo-FC without condensates at the highest expression levels (gray, 18000-20000) are only 368-456, while FC signals of 0.5N FUS-Homo-FC with condensates at the lowest expression levels (green, 4000-5000) are 2500-4700. Considering that relative protein expression levels are similar for scaffold and client proteins under varied protein expression levels (Fig. 3d), 0.5N FUS-Homo-FC levels even inside condensates (only ~2-fold enrichment as shown in Fig. 3c) in the lowest protein expressing cells (green, 4000-5000) could be lower than mCh-0.5N FUS-Homo-FC levels without condensates at the highest expression levels (gray, 18000-20000). These data might suggest that strong probe complementation occurs inside condensates even when inside probe concentration is similar to (or possibly lower than) outside probe concentration.

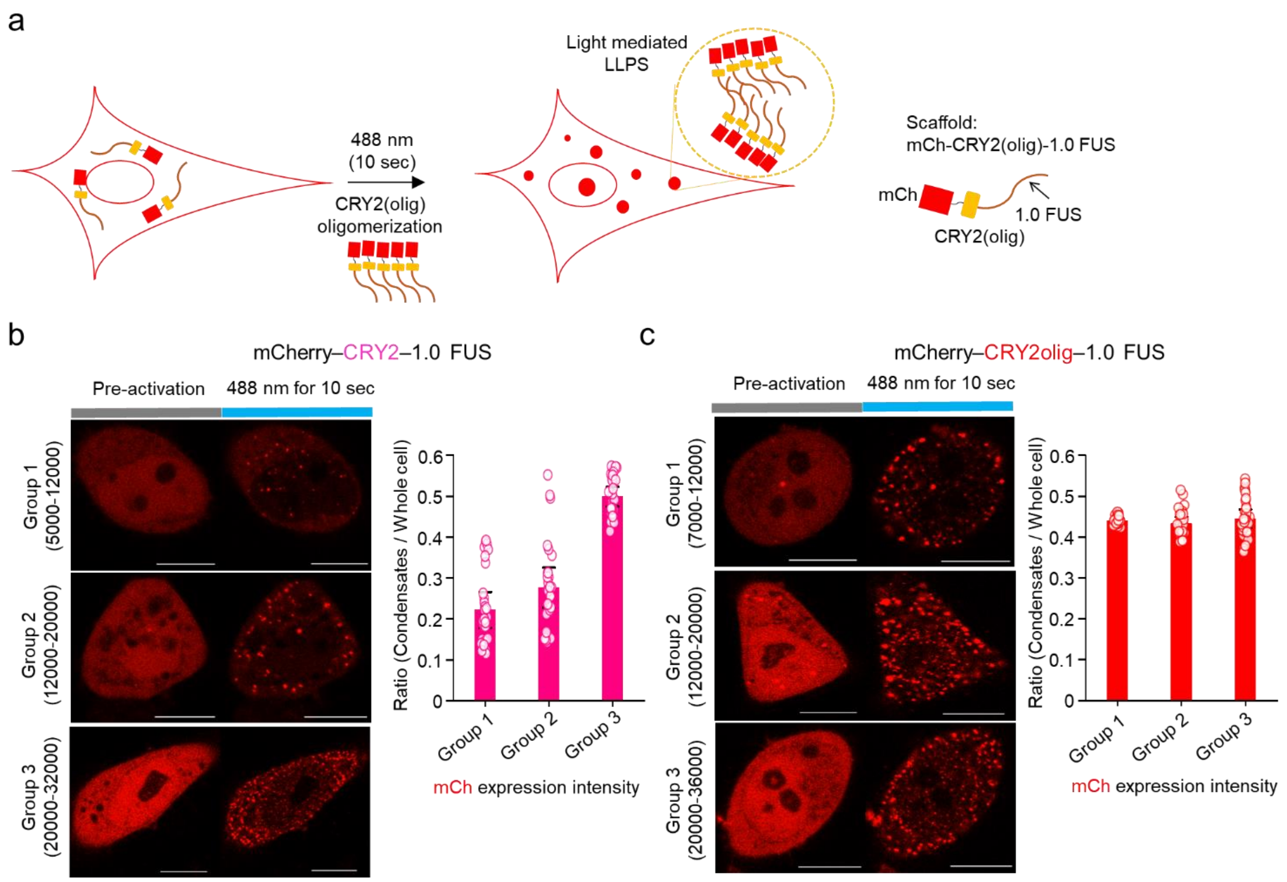

Supplementary Figure 7 Light-induced CRY2-FUS compartment formation in cells. **(a)** Schematic illustration of light-inducible cellular condensate formation of mCh-CRY2(or CRY2olig)-1.0 FUS. Fluorescence images of cells expressing **(b)** mCh-CRY2-1.0 FUS or **(c)** mCh-CRY2olig-1.0 FUS before and after 10 sec 488 nm light activation. Cells are categorized into three groups depending on protein expression levels. mCh-fused scaffold signal ratios between condensates and whole cells are indicated (right) for different groups. Scale bars: 10 µm. Data are presented as mean values with +/- 1 s.d. as error bars (n = 33 cells from three independent experiments).

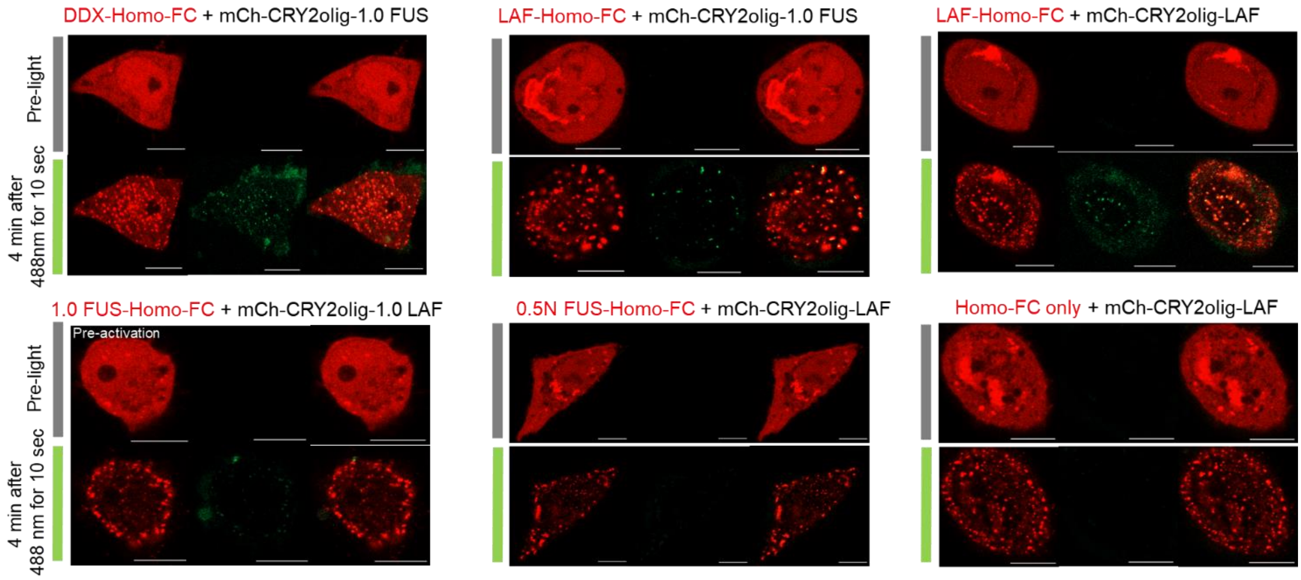

Supplementary Figure 8 Fluorescence images of cells expressing mCh-CRY2olig-1.0 FUS or mCh-CRY2olig-LAF (scaffold) with various IDP-fused Homo-FC clients before and after 10 sec 488 nm light activation and 4 min incubation. Scale bars: 10 µm. Image data for Fig. 4e.

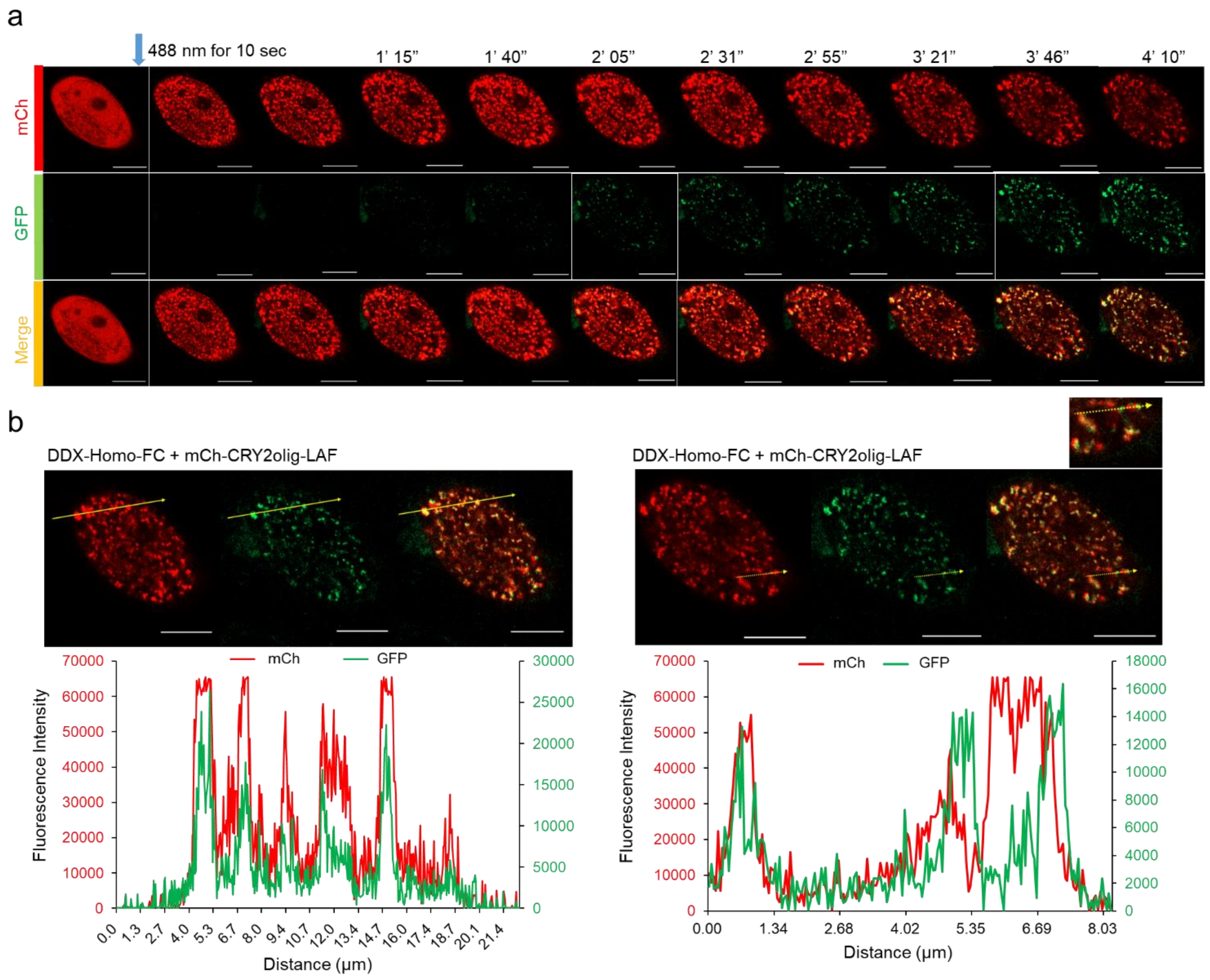

Supplementary Figure 9 Real-time observation of fluorescence complementation inside light-induced LAF condensates. **(a)** Real-time live cell images of mCh-CRY2olig-LAF condensate formation by 10 sec 488 nm light and subsequent DDX-Homo-FC client complementation during 4 min. **(b)** Fluorescence line-profiles of mCh (scaffold: mCh-CRY2olig-LAF) and GFP (client: DDX-Homo-FC) signals. The images are same as Fig. 4d. Most mCh and GFP signals are well-aligned, indicating protein co-localization. A small portion of signals show imperfect co-localization as shown in the right line-profiles. Scale bars: 10 μm.

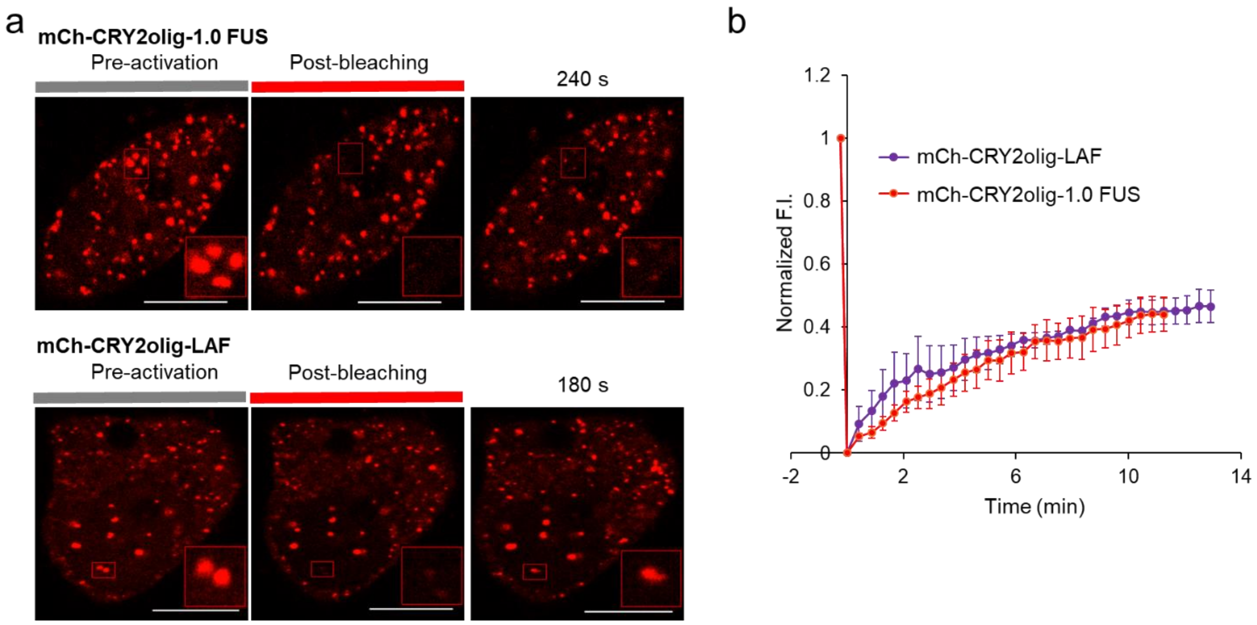

Supplementary Figure 10 FRAP recovery **(a)** images and **(b)** profiles of scaffold proteins of mCh-CRY2olig-1.0 FUS and mCh-CRY2olig-LAF condensates. Scale bars: 10 µm. Data (point) are presented as mean values with +/- 1 s.d. as error bars from three independent experiments.

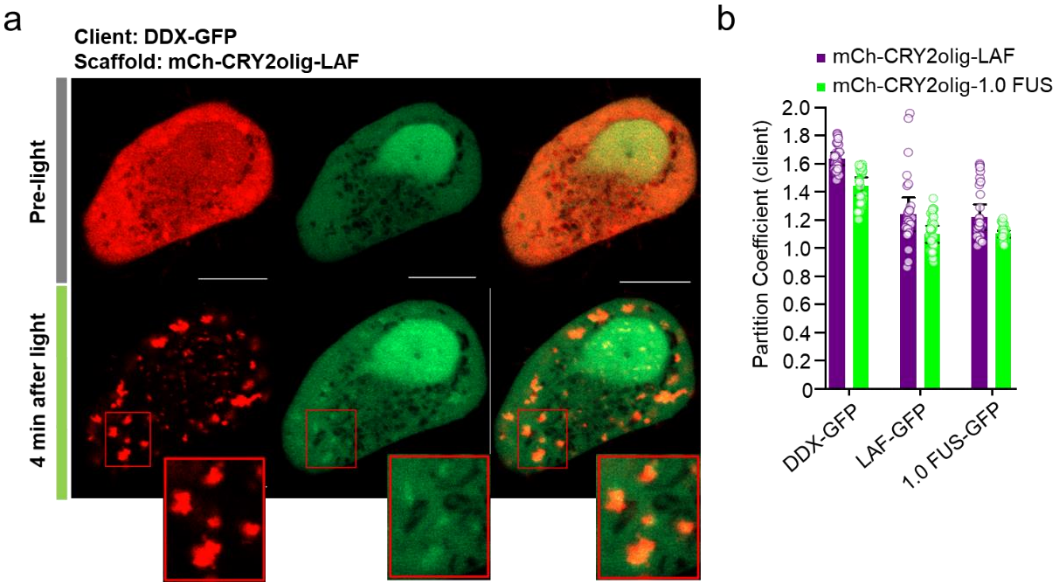

Supplementary Figure 11 IDP-GFP client enrichment into light-induced CRY2olig-IDP condensates. **(a)** Fluorescence images of cells expressing mCh-CRY2olig-LAF (scaffold) with DDX-GFP (client) before and after 10 sec 488 nm light activation and 4 min incubation. Scale bars: 10 µm. **(b)** Partition coefficients of DDX-, LAF, or 1.0 FUS-fused GFP inside and outside mCh-CRY2olig-IDP condensates. Data are presented as mean values with +/- 1 s.d. as error bars (n = 33 cells from three independent experiments).

Before light illumination

	Scaffold	Client	
	mCh-CRY2olig-LAF	DDX-Homo-FC	
Cell number	mCh expression signal	Complementation signal (GFP)	GFP/mCh
1	4045.37	48.59	0.01
2	4169.49	103.90	0.02
3	4789.52	88.47	0.02
4	5501.50	81.04	0.01
5	5816.05	77.92	0.01
6	6367.74	175.42	0.03
7	7511.88	98.83	0.01
8	7554.78	107.78	0.01
9	8197.46	171.74	0.02
10	9352.63	225.17	0.02
11	9666.53	219.93	0.02
12	9864.86	122.36	0.01
13	12014.48	186.58	0.02
14	12068.23	172.79	0.01
15	13797.01	185.09	0.01
16	13985.81	379.52	0.03
17	14038.24	302.08	0.02
18	14164.25	357.77	0.03
19	14363.92	447.22	0.03
20	14951.88	431.68	0.03
21	16325.11	267.99	0.02
22	16981.68	345.28	0.02
23	17032.14	336.80	0.02
24	17167.38	229.65	0.01
25	17301.70	344.92	0.02
26	17416.05	335.42	0.02
27	18078.55	354.58	0.02
28	18131.63	390.80	0.02
29	18357.64	267.20	0.01
30	18720.18	457.86	0.02
31	19517.37	506.20	0.03
32	19744.92	290.04	0.01
33	19881.32	255.77	0.01
AVE			0.02
STDEV			0.01

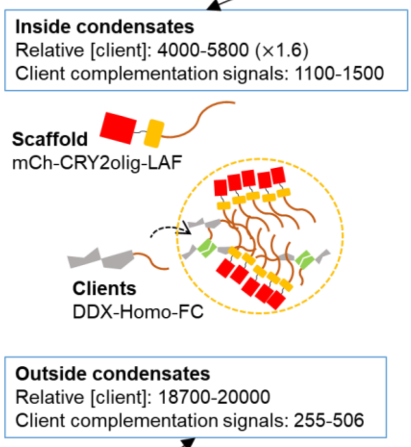

After light illumination

	Scaffold	Client	
	mCh-CRY2olig-LAF	DDX-Homo-FC	
Cell number	mCh expression signal	Complementation signal (GFP)	GFP/mCh
1	4165.82	1150.44	0.28
2	4496.80	1121.00	0.25
3	5107.27	1126.07	0.22
4	5816.56	1523.83	0.26
5	6255.30	1422.80	0.23
6	6638.27	1717.65	0.26
7	6681.90	1311.96	0.20
8	7014.88	1827.99	0.26
9	7408.01	1242.06	0.17
10	7461.65	1421.58	0.19
11	7476.78	1539.53	0.21
12	8195.72	1723.73	0.21
13	8597.10	1467.22	0.17
14	8898.92	2824.41	0.32
15	9158.43	2316.38	0.25
16	10130.76	1709.15	0.17
17	10599.14	2693.96	0.25
18	10961.21	1988.14	0.18
19	11364.53	2611.32	0.23
20	11877.70	3193.37	0.27
21	11994.75	3440.91	0.29
22	13062.48	3215.59	0.25
23	13139.44	2990.55	0.23
24	13885.60	2683.19	0.19
25	14083.52	2498.82	0.18
26	15098.86	3683.89	0.24
27	16595.97	3740.90	0.23
28	17539.86	2752.57	0.16
29	17706.18	2989.29	0.17
30	18709.25	3826.53	0.20
31	19309.55	4087.41	0.21
32	19598.44	4602.97	0.23
33	19736.79	5366.99	0.27
AVE			0.22
STDEV			0.04

Supplementary Figure 12 Protein expression signals (mCh-CRY2olig-LAF scaffold) and FC signals of the DDX-Homo-FC client before or after 10 sec light activation and 4 min incubation for examined 33 cells. Four cells with the highest protein expression (mCh) before light activation (gray) and four cells with the lowest expression (mCh) after light activation (green) are indicated. These values are raw data for the Fig. 4e graph.

Note: Relative DDX-Homo-FC concentrations outside condensates at the highest expression levels (gray, 18700-20000) are estimated based on scaffold expression levels since relative client/scaffold expression levels are mostly constant. Relative DDX-Homo-FC concentrations inside condensates at the lowest expression levels (green, 4000-5800 × 1.6 = 6400-9280) are estimated based on scaffold expression levels and the client enrichment factor (1.6 in Supplementary Fig. 11b). These values clearly indicate that probe complementation is more effective inside condensates than outside even when inside probe concentration is lower than outside.

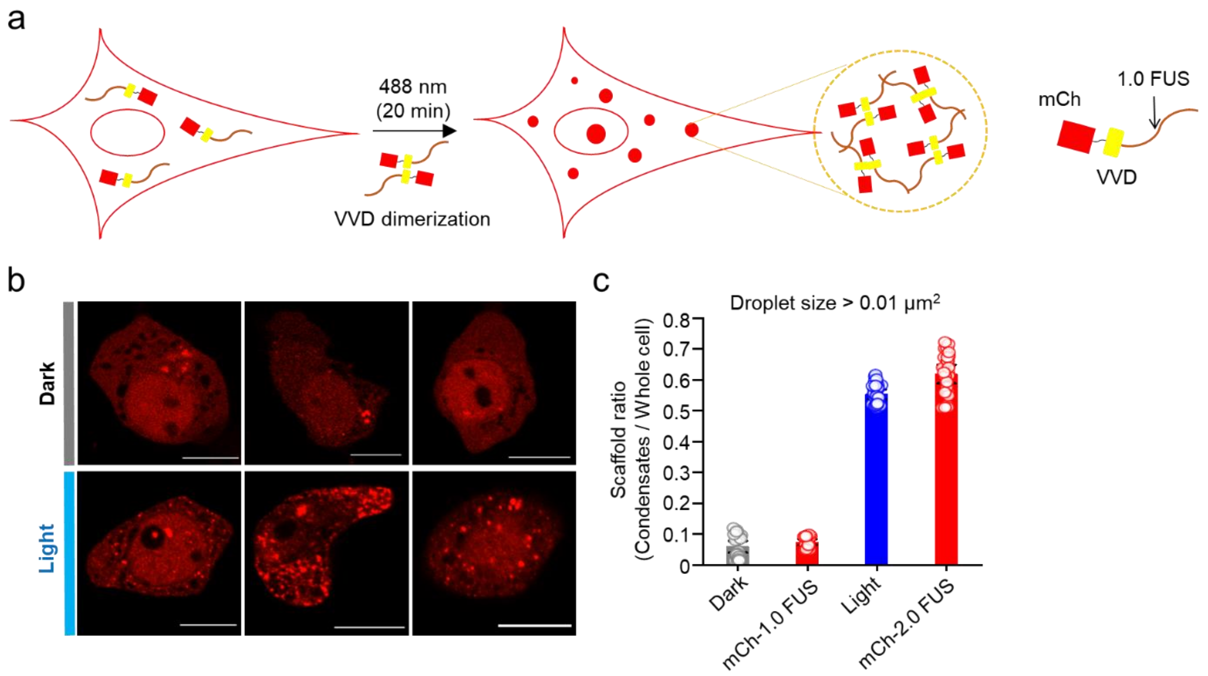

Supplementary Figure 13 Light-induced VVD-FUS compartment formation in cells. **(a)** Schematic illustration of light-inducible cellular condensate formation of mCh-VVD-1.0 FUS. **(b)** Fluorescence images of cells expressing mCh-VVD-1.0 FUS without (dark) or with (Light) 20 min 488 nm light activation. Scale bars: 10 μm. **(c)** mCh-VVD-1.0 FUS signal ratios between condensates and whole cells with or without light activation. For comparison, mCh-scaffold signal ratios between condensates and whole cells for mCh-1.0 FUS and mCh-2.0 FUS (from Fig. 1b) are also included. Data are presented as mean values with +/- 1 s.d. as error bars (n = 33 cells from three independent experiments).

Note: The degree of phase separation for 20 min light-exposed mCh-VVD-1.0 FUS was similar to that of mCh-2.0 FUS. This implies that dimerization effectively increases the IDR length two-fold. On the other hand, in the absence of light, mCh-VVD-1.0 FUS signals were similar to those of mCh-1.0 FUS, showing ineffective condensate formation (Supplementary Fig. 13c).

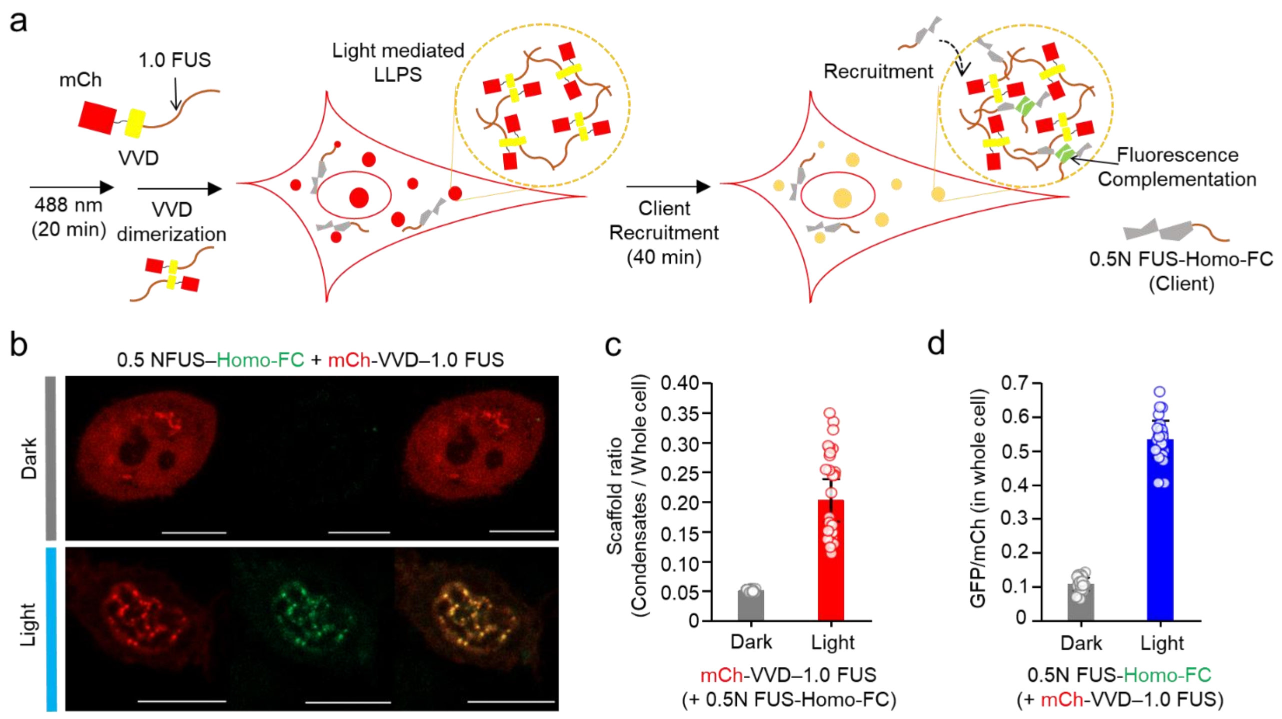

Supplementary Figure 14 Fluorescence complementation of recruited client probes inside light-induced IDP compartments. **(a)** Schematic illustration of light-inducible cellular condensate formation of mCh-VVD-1.0 FUS, 0.5N FUS-Homo-FC recruitment, and subsequent client complementation for (green) fluorescence turn-on. **(b)** Fluorescence images of cells expressing mCh-VVD-1.0 FUS (scaffold) and 0.5N FUS-Homo-FC (client) without (dark) or with (light) 20 min 488 nm light activation. Scale bars: 10 μm. **(c)** Total mCh intensities of condensates divided by whole cell mCh intensities with or without light activation. Data are presented as mean values with +/- 1 s.d. as error bars (n = 33 cells from three independent experiments). **(d)** Total GFP (complemented Homo-FC)-to-mCh ratios for cells with or without light activation. Data are presented as mean values with +/- 1 s.d. as error bars (n = 33 cells from three independent experiments).

Dark

	Scaffold	Client	
	mCh-VVD-1.0 FUS	0.5N FUS Homo-FC	
Cell number	mCh expression signal	Complementation signal (GFP)	GFP/mCh
1	4017.78	407.99	0.10
2	4950.88	598.81	0.12
3	5915.78	785.23	0.13
4	6602.68	665.34	0.10
5	6944.92	603.35	0.09
6	7009.83	719.58	0.10
7	7797.33	908.02	0.12
8	8906.90	846.12	0.09
9	10810.07	1044.66	0.10
10	12075.91	1219.54	0.10
11	12856.58	1294.35	0.10
12	13898.00	1258.69	0.09
13	14027.66	1764.27	0.13
14	14401.11	1286.59	0.09
15	14588.72	1280.58	0.09
16	14649.55	1519.33	0.10
17	15585.63	2242.15	0.14
18	15607.23	2027.48	0.13
19	16669.42	2276.05	0.14
20	16809.02	1505.50	0.09
21	16840.28	1569.93	0.09
22	16904.33	1775.53	0.11
23	17618.73	1149.24	0.07
24	17710.70	2426.92	0.14
25	17865.38	2072.20	0.12
26	18310.36	2422.18	0.13
27	18823.07	1339.75	0.07
28	18965.85	2437.98	0.13
29	19084.66	2045.86	0.11
30	19085.52	2499.12	0.13
31	19165.41	2214.14	0.12
32	19312.71	2263.66	0.12
33	19872.20	2085.96	0.10
AVE			0.11
STDEV			0.02

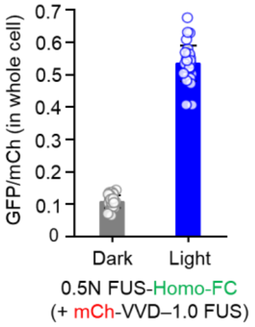

Supplementary Fig. 14d

Light

	Scaffold	Client	
	mCh-VVD-1.0 FUS	0.5N FUS Homo-FC	
Cell number	mCh expression signal	Complementation signal (GFP)	GFP/mCh
1	4211.37	2133.99	0.51
2	4698.88	2721.30	0.58
3	4812.11	2594.15	0.54
4	4932.64	2002.89	0.41
5	5491.58	3091.49	0.56
6	6341.74	3984.02	0.63
7	6478.09	3592.71	0.55
8	6824.96	4149.09	0.61
9	7021.63	3871.95	0.55
10	7344.64	4033.23	0.55
11	7505.31	3764.70	0.50
12	7971.27	4099.45	0.51
13	8169.75	4443.05	0.54
14	8231.73	4489.67	0.55
15	8248.38	4069.54	0.49
16	8753.42	4831.60	0.55
17	9075.00	3689.56	0.41
18	9103.13	4529.46	0.50
19	9784.74	5097.03	0.52
20	10179.20	5309.85	0.52
21	10336.24	5049.76	0.49
22	11691.50	6251.68	0.53
23	12428.72	7833.55	0.63
24	12761.15	6020.57	0.47
25	12859.79	7242.32	0.56
26	16728.78	9092.84	0.54
27	18449.68	8881.65	0.48
28	18612.22	9929.93	0.53
29	18660.86	12597.59	0.68
30	19036.23	9935.68	0.52
31	19336.46	10500.90	0.54
32	19762.25	9954.23	0.50
33	19884.41	11293.16	0.57
AVE			0.53
STDEV			0.06

Supplementary Figure 15 Protein expression signals (mCh-VVD-1.0 FUS scaffold) and FC signals of the 0.5N FUS-Homo-FC client without (Dark) or with 20 min light activation (Light) for examined 33 cells. Four cells with the highest protein expression (mCh) without light activation (gray) and four cells with the lowest expression (mCh) with light activation (green) are indicated. These values are raw data for the Supplementary Fig. 14d graph, which is shown in the middle. Data are presented as mean values with +/- 1 s.d. as error bars (n = 33 cells from three independent experiments).

Note: FC signals of 0.5N FUS-Homo-FC with light-induced condensates at the lowest expression levels (green, 4000-5000) are ~2000-2700. These values are again significantly higher than FC signals of mCh-0.5N FUS-Homo-FC without condensates at the highest expression levels (368-456, from Supplementary Fig. 6a) and comparable to FC signals of 0.5N FUS-Homo-FC without light activation (but with mCh-VVD-1.0 FUS scaffold) at the highest expression levels (gray, 19000-20000).

Free Dronpa

	Scaffold	Client	
	mCh-CRY2olig-LAF	Dronpa	
Cell number	mCh expression signal	Complementation signal (GFP)	GFP/mCh
1	4124.89	320.46	0.08
2	4409.58	160.66	0.04
3	4653.47	214.48	0.05
4	5100.17	294.37	0.06
5	6051.08	539.66	0.09
6	8473.26	801.26	0.09
7	8501.28	662.78	0.08
8	8704.50	868.87	0.10
9	9865.61	531.04	0.05
10	10202.96	470.33	0.05
11	11429.62	627.41	0.05
12	11716.40	538.71	0.05
13	12402.78	781.85	0.06
14	12914.24	1092.33	0.08
15	13146.85	982.62	0.07
16	14009.30	1383.60	0.10
17	14480.33	1034.22	0.07
18	14986.30	1083.81	0.07
19	15104.23	1231.04	0.08
20	15343.60	1112.48	0.07
21	15584.97	1261.28	0.08
22	15638.73	1483.32	0.09
23	15694.31	1304.38	0.08
24	16187.20	1066.29	0.07
25	17137.96	1379.49	0.08
26	17203.07	1687.71	0.10
27	17380.15	1474.00	0.08
28	17491.83	1257.72	0.07
29	18023.16	1373.99	0.08
30	18455.22	1519.72	0.08
31	19154.63	1965.72	0.10
32	19757.79	1483.68	0.08
33	19878.91	1821.02	0.09
AVE			0.08
STDEV			0.02

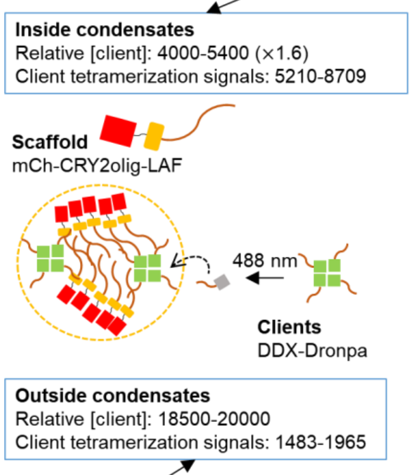

DDX-fused Dronpa

	Scaffold	Client	
	mCh-CRY2olig-LAF	DDX-Dronpa	
Cell number	mCh expression signal	Complementation signal (GFP)	GFP/mCh
1	4021.52	6951.32	1.73
2	4135.96	7698.84	1.86
3	4715.37	5210.78	1.11
4	5409.97	8709.80	1.61
5	5840.97	8962.60	1.53
6	6011.53	9605.99	1.60
7	6369.98	7058.11	1.11
8	6773.99	11691.93	1.73
9	6891.00	8107.54	1.18
10	7680.82	12382.10	1.61
11	7856.11	12493.07	1.59
12	8311.42	13078.34	1.57
13	9053.28	16088.76	1.78
14	9456.55	11128.54	1.18
15	9807.95	18571.04	1.89
16	10050.26	19799.86	1.97
17	10341.44	20094.70	1.94
18	10503.36	15058.14	1.43
19	11008.39	13564.56	1.23
20	11538.00	15977.40	1.38
21	12443.30	16454.02	1.32
22	12899.48	22893.10	1.77
23	13431.14	15985.30	1.19
24	13827.97	20944.87	1.51
25	14232.06	18839.45	1.32
26	16175.27	18838.08	1.16
27	17704.56	21876.28	1.24
28	17922.79	28950.27	1.62
29	18107.04	32984.85	1.82
30	18806.46	22218.44	1.18
31	19369.07	27129.24	1.40
32	19509.82	34896.74	1.79
33	19747.13	38602.62	1.95
AVE			1.52
STDEV			0.28

Supplementary Figure 16 Protein expression signals (mCh-CRY2olig-LAF scaffold) and tetramerization signals of the DDX-Dronpa client before or after 10 sec light activation and 4 min incubation for examined 33 cells. Four cells with the highest protein expression (mCh) with free Dronpa (gray) and four cells with the lowest expression (mCh) with DDX-fused Dronpa (green) are indicated. These values are raw data for the Fig. 5d graph.

Note: Relative Dronpa concentrations outside condensates at the highest expression levels (gray, 18500-20000) are estimated based on scaffold expression levels. Relative DDX-Dronpa concentrations inside condensates at the lowest expression levels (green, 4000-5400 × 1.6 = 6400-8640) are estimated based on scaffold expression levels and the client enrichment factor (1.6 in Supplementary Fig. 11b). Again, these values clearly indicate that Dronpa tetramerization is more effective inside condensates than outside even when inside probe concentration is lower than outside.

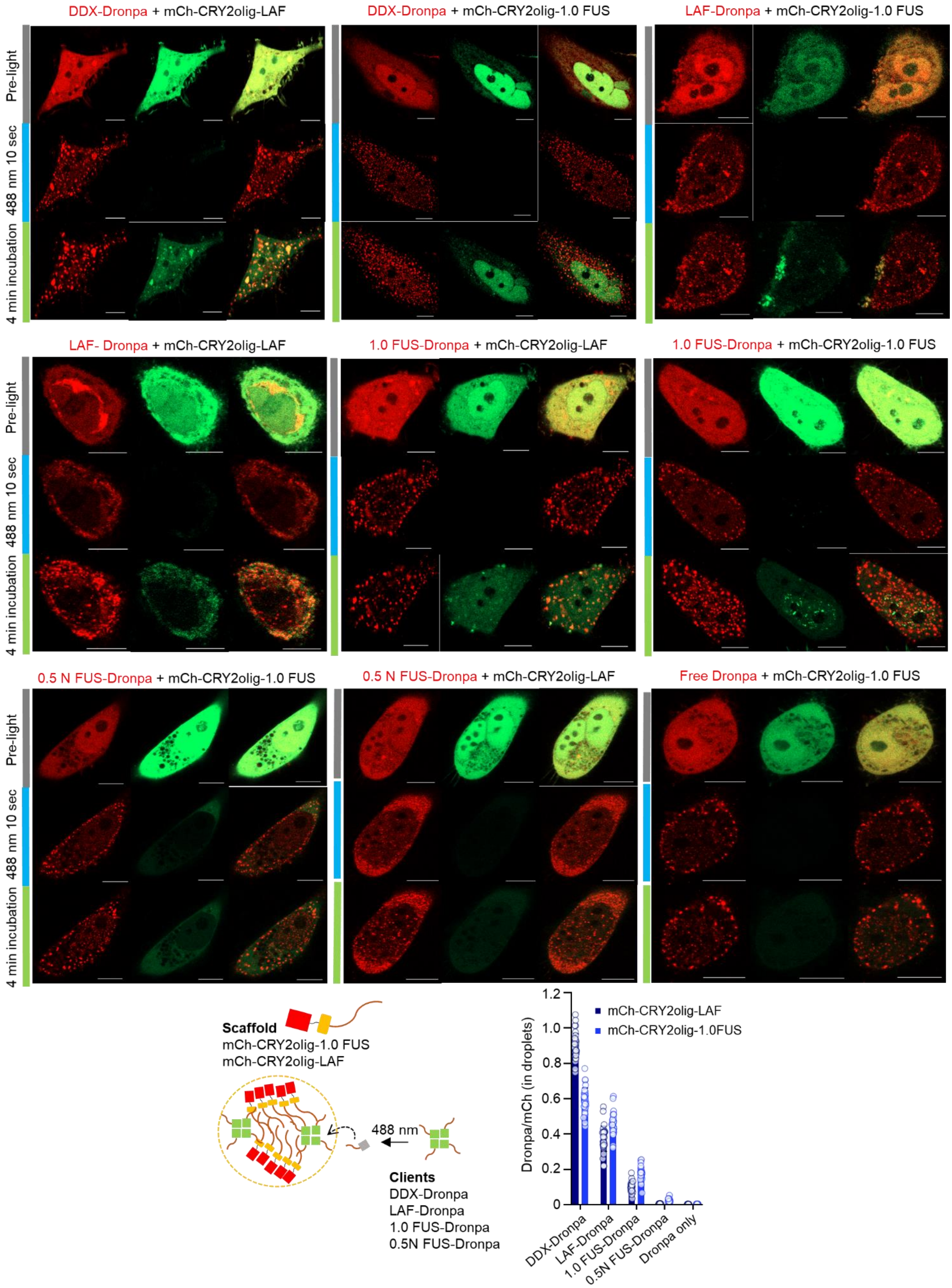
DDX-Dronpa + mCh-CRY2olig-LAF
DDX-Dronpa + mCh-CRY2olig-1.0 FUS
LAF-Dronpa + mCh-CRY2olig-1.0 FUS
LAF- Dronpa + mCh-CRY2olig-LAF
1.0 FUS-Dronpa + mCh-CRY2olig-LAF
1.0 FUS-Dronpa + mCh-CRY2olig-1.0 FUS
0.5 N FUS-Dronpa + mCh-CRY2olig-1.0 FUS
0.5 N FUS-Dronpa + mCh-CRY2olig-LAF
Free Dronpa + mCh-CRY2olig-1.0 FUS
Pre-light
488 nm 10 sec
4 min incubation
Scaffold
mCh-CRY2olig-1.0 FUS
mCh-CRY2olig-LAF
488 nm
Clients
DDX-Dronpa
LAF-Dronpa
1.0 FUS-Dronpa
0.5N FUS-Dronpa
Dronpa/mCh (in droplets)
1.2
1.0
0.8
0.6
0.4
0.2
0
mCh-CRY2olig-LAF
mCh-CRY2olig-1.0FUS
DDX-Dronpa
LAF-Dronpa
1.0 FUS-Dronpa
0.5N FUS-Dronpa
Dronpa only

Supplementary Figure 17 Fluorescence images of cells expressing mCh-CRY2olig-1.0 FUS or mCh-CRY2olig-LAF (scaffold) with various IDP-fused Dronpa clients before and after 10 sec 488 nm light activation and 4 min incubation. Scale bars: 10 µm. Image data for Fig. 5d. Schematic illustration of light-inducible cellular condensates with two kinds of scaffolds and four different Dronpa clients with various IDPs is shown below. Total GFP(tetramerized Dronpa)-to-mCh ratios for condensates (right) with various combinations of IDP scaffolds and clients is shown. Data are presented as mean values with +/- 1 s.d. as error bars (n = 33 cells from three independent experiments).

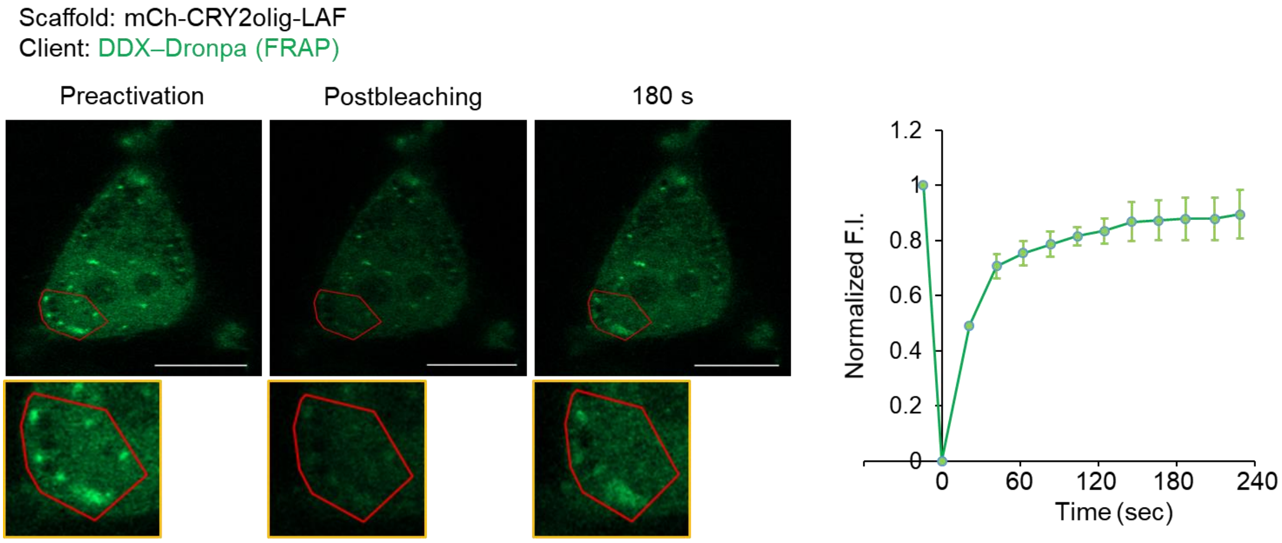

Supplementary Figure 18 FRAP recovery images and profiles of client DDX-Dronpa proteins around mCh-CRY2olig-LAF condensates. Scale bars: 10 µm. Data (point) are presented as mean values with +/- 1 s.d. as error bars from three independent experiments.

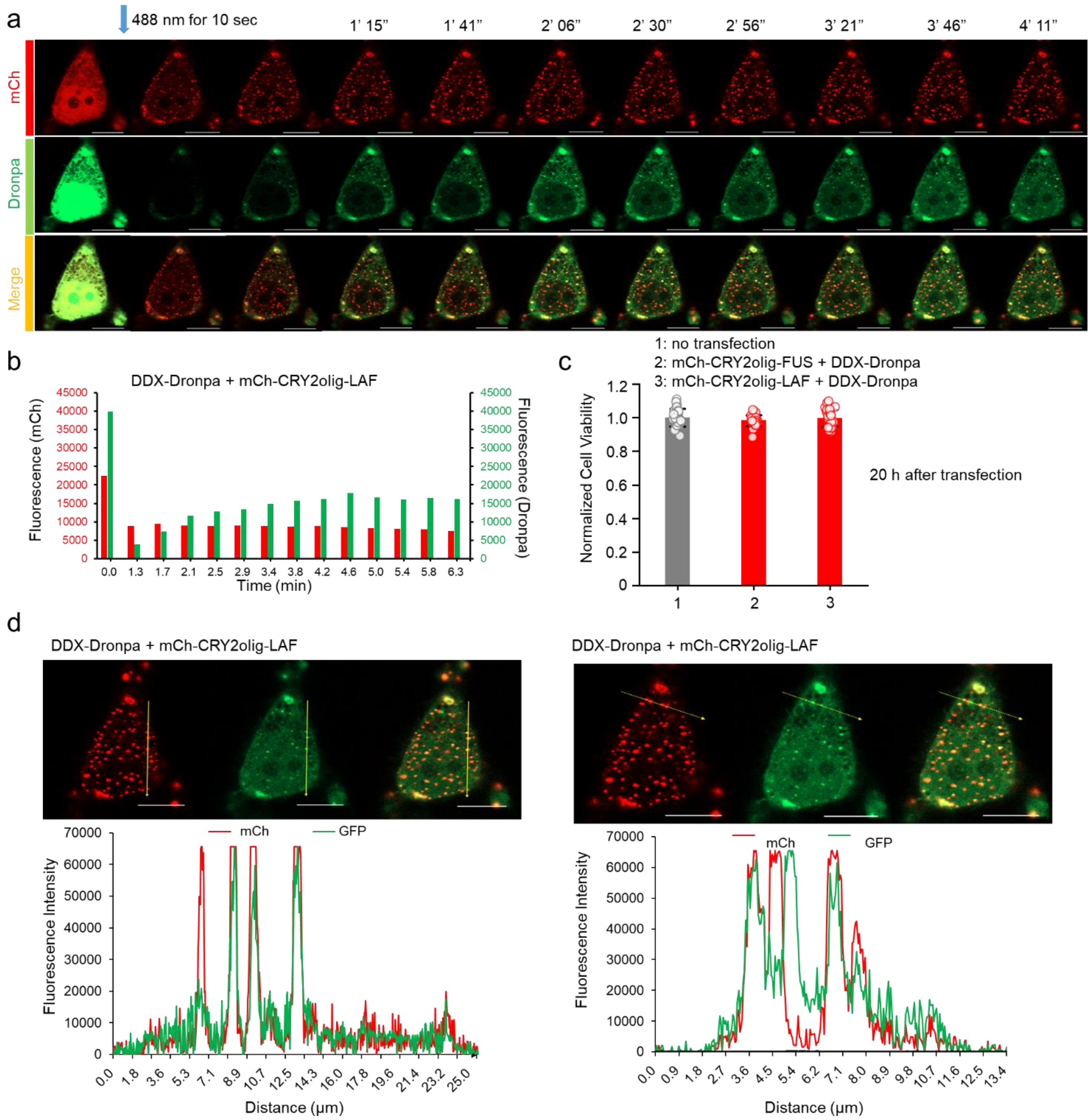

Supplementary Figure 19 Real-time observation of fluorescence protein interactions inside light-induced LAF condensates. **(a)** Real-time live cell images of mCh-CRY2olig-LAF condensate formation by 10 sec 488 nm light and subsequent DDX-Dronpa client tetramerization (turn-on) during 4 min. **(b)** Scaffold (mCh) and client tetramerization (Dronpa) signal changes inside condensates at various time point after 10 sec light activation. **(c)** Cell viabilities of Hela cells transfected with a mCh-CRY2olig-IDR scaffold and a DDX-Dronpa client at 20 h after transfection. Data are presented as mean values with +/- 1 s.d. as error bars (n = 30 wells from three independent experiments). **(d)** Fluorescence line-profiles of mCh (scaffold: mCh-CRY2olig-LAF) and GFP (client: DDX-Dronpa) signals. The images are same as Fig. 5b. Most mCh and GFP signals are well-aligned, indicating

protein co-localization. A small portion of signals show imperfect co-localization as shown in the right line-profiles. Scale bars: 10 μm.

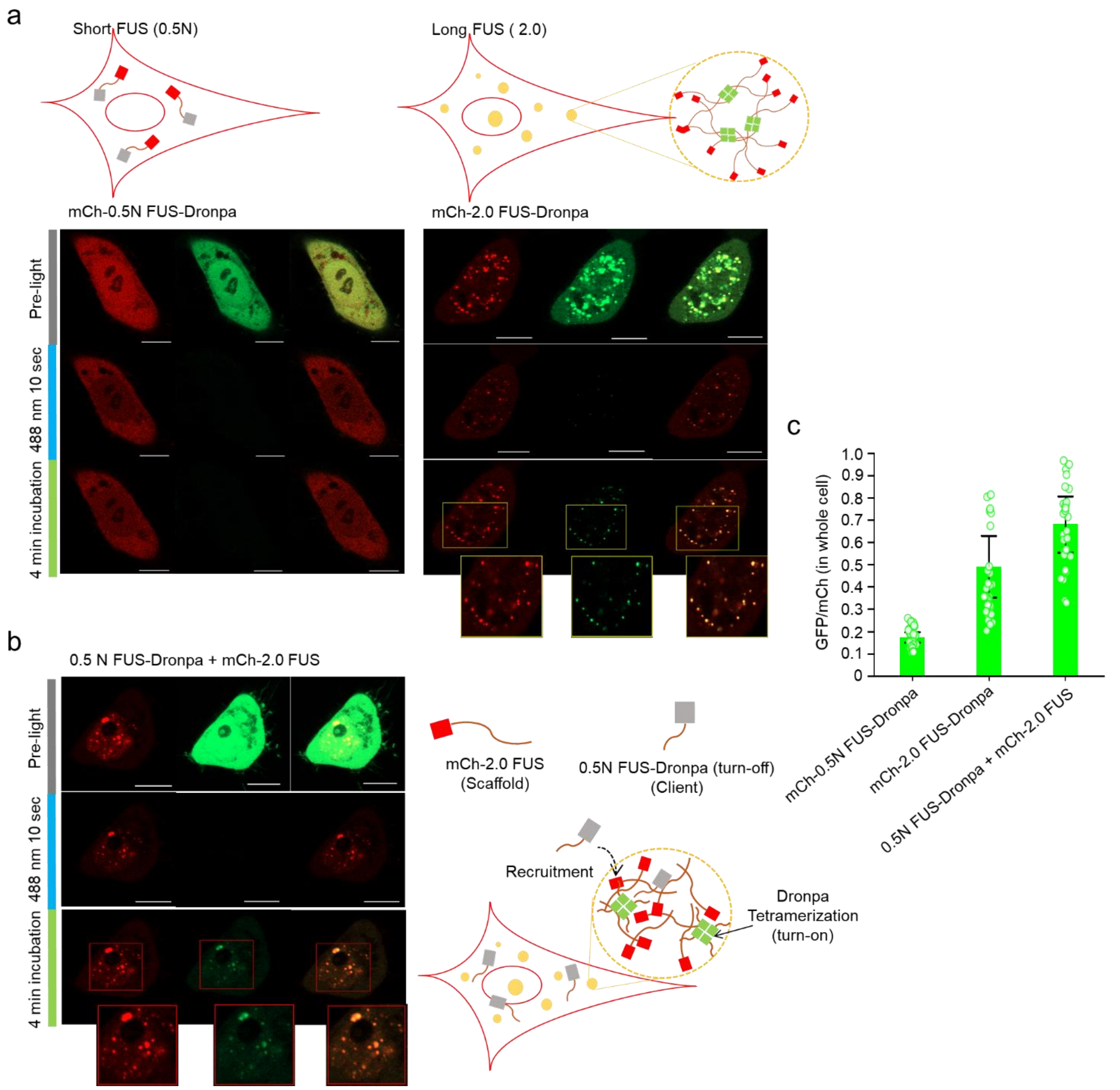

Supplementary Figure 20 Tetramerization of monomerized Dronpa inside repeated-IDP compartments. **(a)** Fluorescence (mCh and Dronpa) images of cells expressing mCh-0.5 FUS-Dronpa or mCh-2.0 FUS-Dronpa (scaffold) before and after 10 sec 488 nm light activation, followed by 4 min incubation. **(b)** Fluorescence (mCh and Dronpa) images of cells co-expressing 0.5N FUS-Dronpa (client) and mCh-2.0 FUS (scaffold) before and after 10 sec 488 nm light activation, followed by 4 min incubation. Scale bars: 10 μm. **(c)** Total GFP(tetramerized Dronpa)-to-mCh ratios in whole cells. Data are presented as mean values with +/- 1 s.d. as error bars (n = 33 cells from three independent experiments).

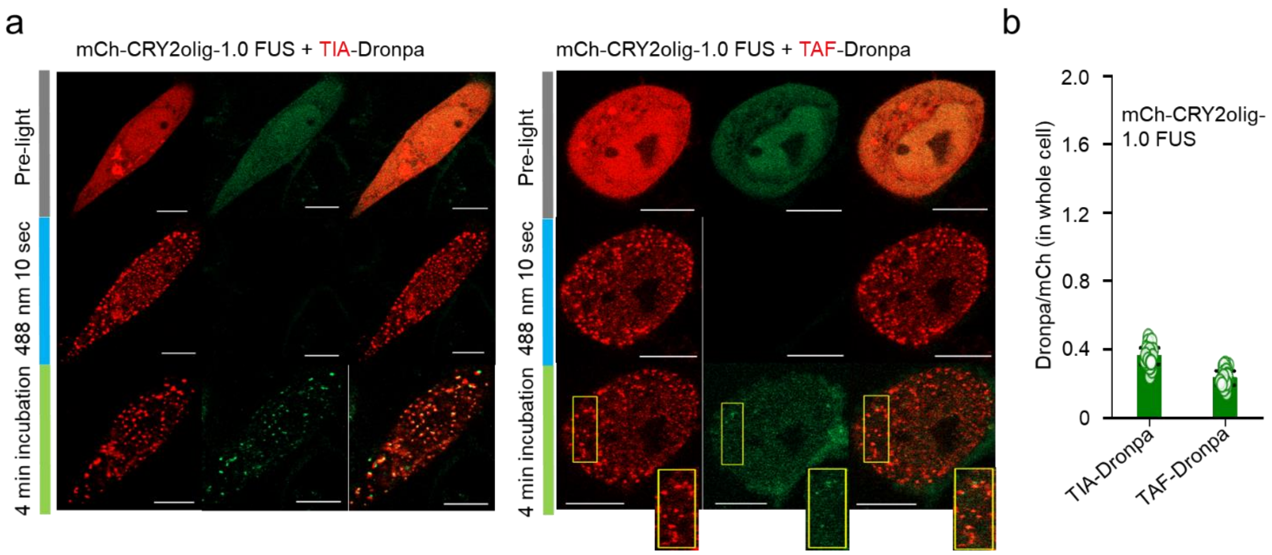

Supplementary Figure 21 TIA- or TAF-fused Dronpa interactions inside light-induced FUS compartments. **(a)** Fluorescence images of cells expressing mCh-CRY2olig-FUS (scaffold) with client TIA-Dronpa (left) or TAF-Dronpa (right) before and after 10 sec 488 nm light activation and 4 min incubation. Scale bars: 10 µm. **(b)** Total tetramerized Dronpa-to-mCh ratios for whole cells with mCh-CRY2olig-1.0 FUS and TIA, TAF-Dronpa clients. Data are presented as mean values with +/- 1 s.d. as error bars (n = 33 cells from three independent experiments).

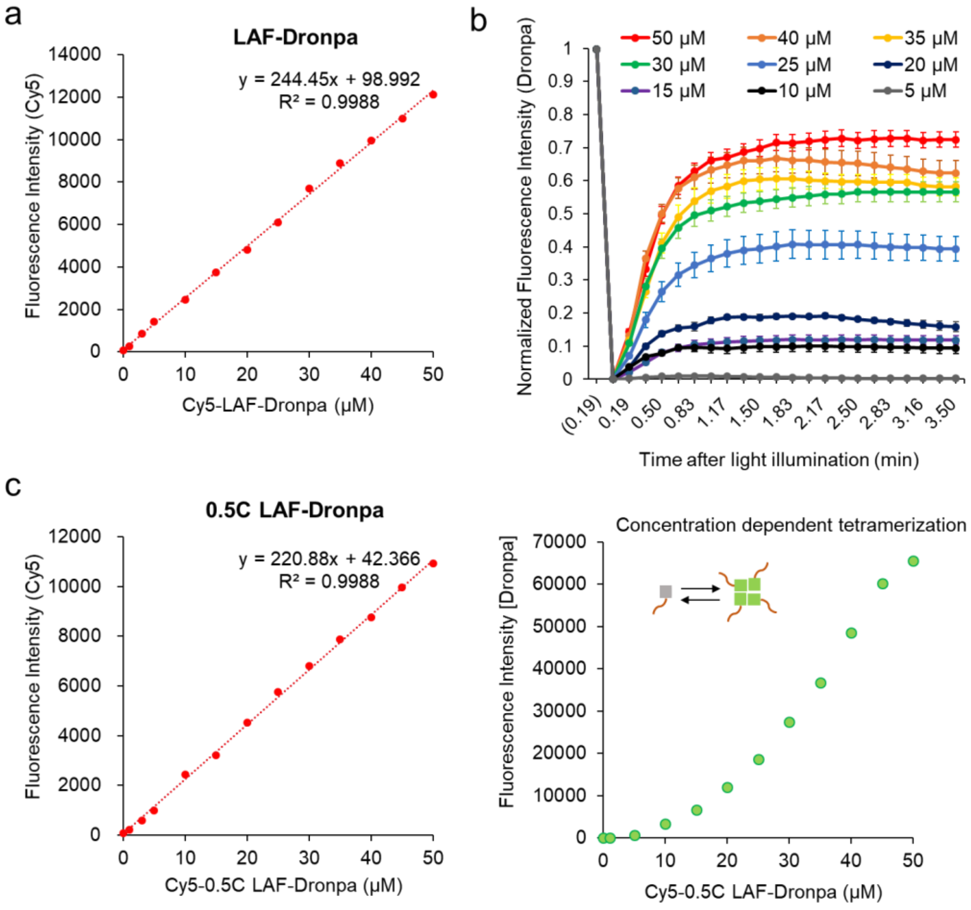

Supplementary Figure 22 Quantitative measurement of Dronpa concentration and tetramerization inside IDR compartments **(a)** Cy5 intensities of Cy5-fused LAF-Dronpa as a function of Dronpa concentration. The linear fit equation is indicated. **(b)** Time-dependent Dronpa turn-on intensities by tetramerization at various monomerized Dronpa concentrations. Data are presented as mean values with +/- 1 s.d. as error bars from three independent experiments. **(c)** Cy5 intensities of Cy5-fused 0.5C LAF-Dronpa as a function of Dronpa concentration (left) and Dronpa fluorescence signals of concentration-varied 0.5C LAF-Dronpa after 10 sec 488 nm light activation for Dronpa monomerization and 4 min incubation for concentration-dependent tetramerization.

Note: Proximity signals of LAF-Dronpa (as well as 0.5C LAF-Dronpa) were reliably measured from 1 µM and up to 50 µM at a fixed imaging condition (i.e. excitation laser power, expose time). Sensitivity-wise, the Dronpa signals at 1 µM was over two-fold higher than that at 0 µM, indicating 1 µM as the limit of detection (see Source Data). However, the proximity range (up to 50 µM) could not be further extended to higher [LAF-Dronpa], since LAF-Dronpa became rather unstable at higher concentrations.

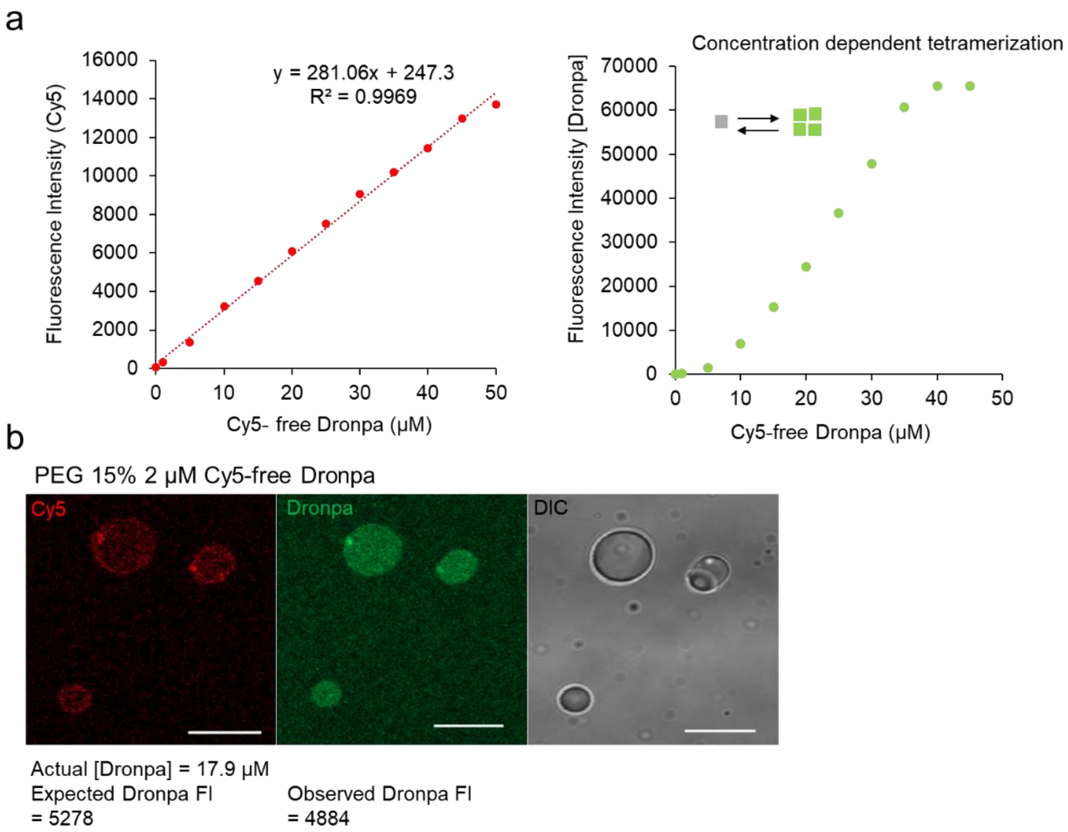

Supplementary Figure 23 Quantitative measurement of free Dronpa concentration and tetramerization inside LAF compartments **(a)** Cy5 intensities of Cy5-fused free Dronpa as a function of Dronpa concentration (left) and Dronpa fluorescence signals of concentration-varied free Dronpa after 10 sec 488 nm light activation for Dronpa monomerization and 4 min incubation for concentration-dependent tetramerization. **(b)** Cy5 and Dronpa fluorescence images of LAF droplets with mixed 2 μM client free Dronpa. A calculated actual (Cy5) client concentration and expected/observed Dronpa fluorescent intensities (FI) are indicated below the images. Scale bars: 10 μm.

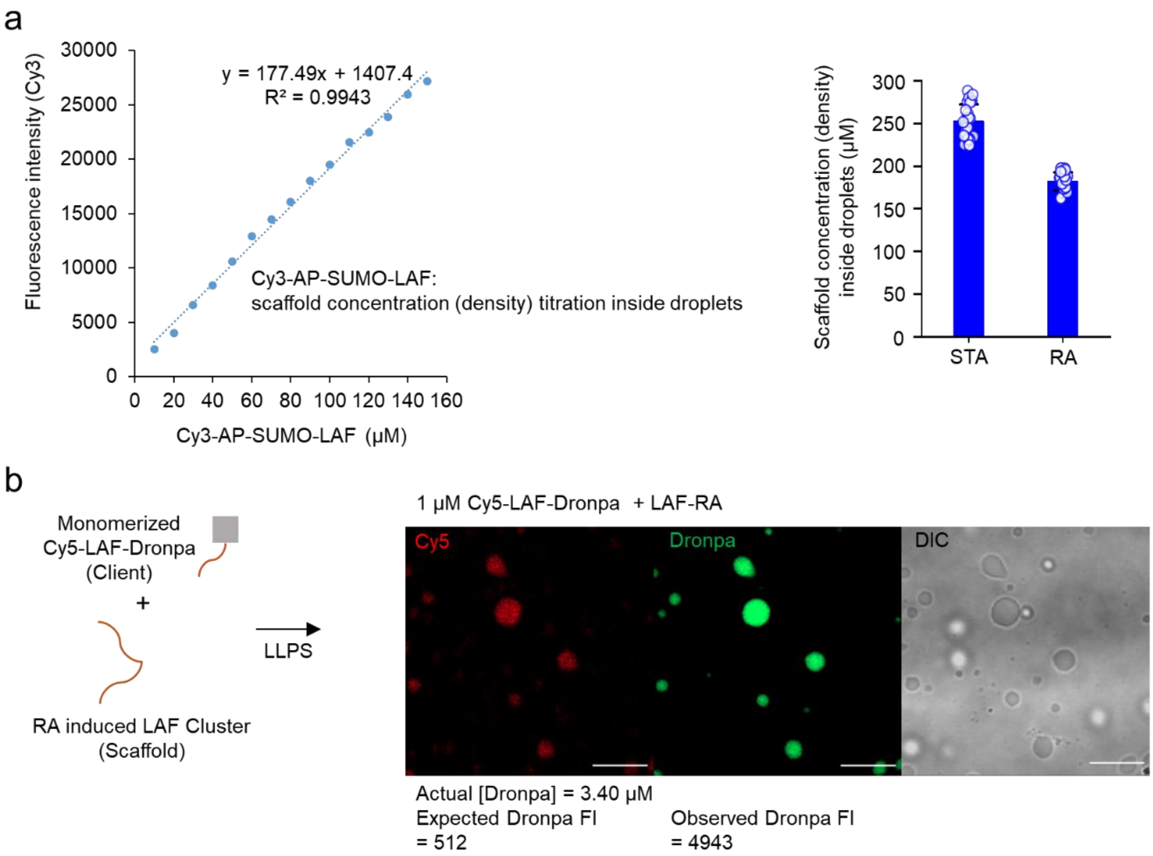

Supplementary Figure 24 Scaffold density determination and quantitative measurement of Dronpa concentration/tetramerization inside LAF-RA compartments **(a)** Scaffold density determination. Cy3 intensities of Cy3-fused AP-SUMO-LAF as a function of scaffold concentration are shown in the left graph. The linear fit equation is indicated. Cy3 signals inside LAF-STA or LAF-RA droplets were measured to calculate LAF concentrations (densities) inside droplets (right graph). Data are presented as mean values with +/- 1 s.d. as error bars (n = 33 droplets from three independent experiments). **(b)** Cy5 and Dronpa fluorescence images of LAF-RA droplets with mixed 1 µM client Cy5-LAF-Dronpa. A calculated actual (Cy5) client concentration and expected/observed Dronpa fluorescent intensities (FI) are indicated below the images. Scale bars: 10 µm.

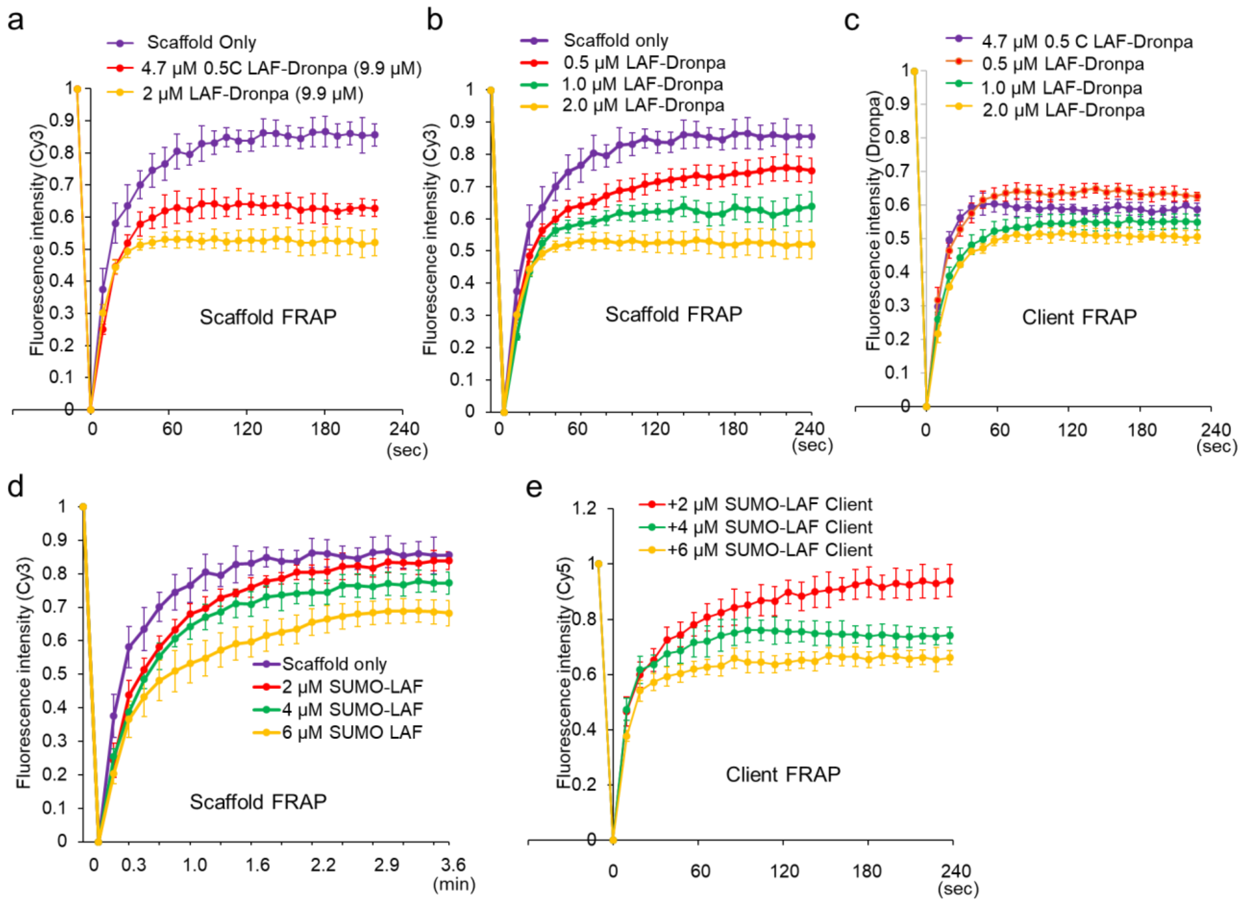

Supplementary Figure 25 Diffusivity changes of LAF droplets by recruited clients. **(a)** FRAP recovery profiles of Cy3-AP-SUMO-LAF (scaffold) condensates with the same concentration (9.9 μM) of LAF-Dronpa or 0.5C LAF-Dronpa (client). **(b)** FRAP recovery profiles of Cy3-AP-SUMO-LAF (scaffold) condensates with increasing concentrations (0.5, 1.0, to 2.0 μM) of LAF-Dronpa (client). **(c)** FRAP recovery profiles of varying concentrations of LAF-Dronpa or 0.5C LAF-Dronpa (client) inside AP-SUMO-LAF (scaffold) condensates. **(d)** FRAP recovery profiles of Cy3-AP-SUMO-LAF (scaffold) condensates with increasing concentrations (2, 4, to 6 μM) of SUMO-LAF (client).). **(e)** FRAP recovery profiles of varying concentrations of SUMO-LAF (client) inside AP-SUMO-LAF (scaffold) condensates. All data (point) are presented as mean values with +/- 1 s.d. as error bars (n = 33 droplets from three independent experiments).

Supplementary Table 1 Normalized GFP signals of the Homo-FC or Dronpa probe from examined scaffold and client cellular combination.

	GFP / mCh (Whole Cell)
Scaffold fused probe	
mCh-0.5N FUS-HomoFC	0.02 ± 0.002
mCh-0.5C FUS-HomoFC	0.01 ± 0.007
mCh-0.75 FUS-HomoFC	0.15 ± 0.13
mCh-1.0 FUS-HomoFC	0.21 ± 0.03
mCh-1.25 FUS-HomoFC	0.41 ± 0.07
mCh-1.5 FUS-HomoFC	0.53 ± 0.15
mCh-2.0 FUS-HomoFC	0.76 ± 0.19
Client-probe + scaffold	
0.5N FUS-Homo-FC + mCh-1.5 FUS	0.49 ± 0.12
0.5N FUS-Homo-FC + mCh-2.0 FUS	0.71 ± 0.19
DDX-Homo-FC + mCh-CRY2olig-LAF	0.22 ± 0.04
DDX-Homo-FC + mCh-CRY2olig-1.0 FUS	0.18 ± 0.02
LAF-Homo-FC + mCh-CRY2olig-LAF	0.12 ± 0.03
LAF-Homo-FC + mCh-CRY2olig-1.0 FUS	0.13 ± 0.02
1.0 FUS Homo-FC + mCh-CRY2olig-LAF	0.08 ± 0.01
1.0 FUS Homo-FC + mCh-CRY2olig-1.0 FUS	0.09 ± 0.01
0.5 N FUS Homo-FC + mCh-CRY2olig-LAF	0.04 ±0.01
0.5 N FUS Homo-FC + mCh-CRY2olig-1.0 FUS	0.04 ± 0.002
Homo-FC only + mCh-CRY2olig-LAF	0.03 ± 0.01
Homo-FC only + mCh-CRY2olig-1.0 FUS	0.03 ± 0.007
DDX-Dronpa + mCh-CRY2olig-LAF	1.52 ± 0.28
DDX-Dronpa + mCh-CRY2olig-1.0 FUS	1.18 ± 0.16
LAF-Dronpa + mCh-CRY2olig-LAF	0.81 ± 0.16
LAF-Dronpa + mCh-CRY2olig-1.0 FUS	1.12 ± 0.13
1.0 FUS Dronpa + mCh-CRY2olig-LAF	0.75 ± 0.12
1.0 FUS Dronpa + mCh-CRY2olig-1.0 FUS	0.77 ± 0.14
0.5 N FUS Dronpa + mCh-CRY2olig-LAF	0.15 ± 0.02
0.5 N FUS Dronpa + mCh-CRY2olig-1.0 FUS	0.22 ± 0.05
Dronpa only + mCh-CRY2olig-LAF	0.08 ± 0.02
Dronpa only + mCh-CRY2olig-1.0 FUS	0.09 ± 0.02
TAF-Dronpa + mCh-CRY2olig-1.0 FUS	0.23 ± 0.04

Supplementary Table 2 Primer Sequences

**Name**	**Primer Sequence** (5' → 3')
FUS_Forward	GCC TCA AAC GAT TAT ACC CAA CAA GCA ACC
FUS_Reverse	TCC ACG GTC CTG CTG TCC ATA GCC
LAF_Forward	GAA AGT AAC CAA TCG AAC AAT GGA GGC AGT GGT
LAF-Reverse	GCC ATC GCC TCC ATT ATC CCG ACG ATC ATT ATT
DDX_Forward	GGA GAT GAA GAT TGG GAA GCA GAA ATC AAC CCT
DDX_Reverse	ACT TTC TCC TCC TTC TGC TTC TGA CTT CCA AGA ATT
TAF15_Forward	TCG GAT TCT GGA AGT TAC GGT CAG TCT
TAF15_Reverse	GCC ACC TCT ATC ACC ACC ACT TGA TCC
TIA1_Forward	ATA AAT CCC GTG CAA CAG CAG AAT CAA ATT GGA
TIA1_Reverse	CTG GGT TTC ATA CCC TGC CAC TCG ATA
CRY2_WT (CRY2PHR 1-498)_Forward	AAG ATG GAC AAA AAG ACT ATA GTT TGG TTT AGA AGA GAC CTA
CRY2_WT (CRY2PHR 1-498)_Reverse	TGC TGC TCC GAT CAT GAT CTG TGC TTC ACG
CRY2olig_Forward	AAG ATG GAC AAA AAG ACT ATA GTT TGG TTT AGA AGA GAC CTA
CRY2olig_Reverse	TGC TGC TCC GAT CAT GAT CTG TGC GCC
Dronpa 145N_Forward	AGT GTG ATT AAA CCA GAC ATG AAG ATC AAG CTG
Dronpa 145N_Reverse	CTT GGC CTG CCT CGG CAG CTC AGA
WT VVD_Forward	CAC ACC CTT TAT GCG CCT GGT GGG TAT
WT VVD_Reverse	TTC AGT TTC ACA CTG AAA TCC CAT GCT ATA ACG
mCherry_Forward	GTG AGC AAG GGC GAG GAG GAC AAT ATG
mCherry_Reverse	CTT GTA CAG CTC GTC CAT GCC GCC
-15sfGFP_Forward	AAA GGA GAA GAA CTT TTC ACT GGA GTT GTC
-15sfGFP_Reverse	GGT AAT GCC CGC CGC ATT CAC ATA TTC
-15sfGFP based Homo-FC_Forward	GCA GAC AAG CAG GAA AAT GGG ATT AAA GCC
-15 sfGFP based Homo-FC_Reverse	AGT GAT ATA CAC GTC ATG GGA GTT AAA ATT GTA CTC

Protein Sequences

WT FUS IDR (1-214): 1.0 FUS
MASNDYTQQATQSYGAYPTQPGQGYSQQSSQPYGQQSYSGYSQSTDTSGYGQSSYSSYGQSQNTGYG
TQSTPQGYGSTGGYGSSQSSQSSYGQQSSYPGYGQQPAPSSTSGSYGSSSQSSSYGQPQSGSYSQQP
SYGGQQQSYGQQQSYNPPQGYGQQNQYNSSSGGGGGGGGGNYGQDQSSMSSGGGSGGGYGNQDQSG
GGGSGGYGQQDRG
(Blue (1-106): 0.5N FUS, Black (107-214): 0.5C FUS)

0.75 FUS (1-156)
MASNDYTQQATQSYGAYPTQPGQGYSQQSSQPYGQQSYSGYSQSTDTSGYGQSSYSSYGQSQNTGYG
TQSTPQGYGSTGGYGSSQSSQSSYGQQSSYPGYGQQPAPSSTSGSYGSSSQSSSYGQPQSGSYSQQP
SYGGQQQSYGQQQSYNPPQGYG

1.25 FUS (1.0 FUS + 0.25N FUS)
MASNDYTQQATQSYGAYPTQPGQGYSQQSSQPYGQQSYSGYSQSTDTSGYGQSSYSSYGQSQNTGYG
TQSTPQGYGSTGGYGSSQSSQSSYGQQSSYPGYGQQPAPSSTSGSYGSSSQSSSYGQPQSGSYSQQP
SYGGQQQSYGQQQSYNPPQGYGQQNQYNSSSGGGGGGGGGNYGQDQSSMSSGGGSGGGYGNQDQSG
GGGSGGYGQQDRGASNDYTQQATQSYGAYPTQPGQGYSQQSSQPYGQQSYSGYSQSTDTSGYGQSS

1.5 FUS (1.0 FUS + 0.5N FUS)
MASNDYTQQATQSYGAYPTQPGQGYSQQSSQPYGQQSYSGYSQSTDTSGYGQSSYSSYGQSQNTGYG
TQSTPQGYGSTGGYGSSQSSQSSYGQQSSYPGYGQQPAPSSTSGSYGSSSQSSSYGQPQSGSYSQQP
SYGGQQQSYGQQQSYNPPQGYGQQNQYNSSSGGGGGGGGGNYGQDQSSMSSGGGSGGGYGNQDQSG
GGGSGGYGQQDRGASNDYTQQATQSYGAYPTQPGQGYSQQSSQPYGQQSYSGYSQSTDTSGYGQSSY
SSYGQSQNTGYGTQSTPQGYGSTGGYGSSQSSQSSYGQQSSYPGYGQQPAP

2.0 FUS (1.0 FUS X 2)
MASNDYTQQATQSYGAYPTQPGQGYSQQSSQPYGQQSYSGYSQSTDTSGYGQSSYSSYGQSQNTGYG
TQSTPQGYGSTGGYGSSQSSQSSYGQQSSYPGYGQQPAPSSTSGSYGSSSQSSSYGQPQSGSYSQQP
SYGGQQQSYGQQQSYNPPQGYGQQNQYNSSSGGGGGGGGGNYGQDQSSMSSGGGSGGGYGNQDQSG
GGGSGGYGQQDRGASNDYTQQATQSYGAYPTQPGQGYSQQSSQPYGQQSYSGYSQSTDTSGYGQSSY
SSYGQSQNTGYGTQSTPQGYGSTGGYGSSQSSQSSYGQQSSYPGYGQQPAPSSTSGSYGSSSQSSSY
GQPQSGSYSQQPSYGGQQQSYGQQQSYNPPQGYGQQNQYNSSSGGGGGGGGGNYGQDQSSMSSGGG
SGGGYGNQDQSGGGGSGGYGQQDRG

AP-SUMO-FUS
GLNDIFEAQKIEWHEMSDSEVNQEAKPEVKPEVKPETHINLKVSDGSSEIFFKIKKTTPLRRLMEAF
AKRQGKEMDSLRFLYDGIRIQADQTPEDLDMEDNDIIEAHREQIGGATYEFASNDYTQQATQSYGAY
PTQPGQGYSQQSSQPYGQQSYSGYSQSTDTSGYGQSSYSSYGQSQNTGYGTQSTPQGYGSTGGYGSS
QSSQSSYGQQSSYPGYGQQPAPSSTSGSYGSSSQSSSYGQPQSGSYSQQPSYGGQQQSYGQQQSYNP
PQGYGQQNQYNSSSGGGGGGGGGNYGQDQSSMSSGGGSGGGYGNQDQSGGGGSGGYGQQDRG
(Yellow: Biotinylation peptide AP tag, Blue: SUMO, Underline: FUS IDR)

mCherry-linker-FUS-linker-Homo-FC

MVSKGEEDNMAIIKEFMRFKVHMEGSVNGHEFEIEGEGEGRPYEGTQTAKLKVTKGGPLPFAWDILS
PQFMYGSKAYVKHPADIPDYLKLSFPEGFKWERVMNFEDGGVVTVTQDSSLQDGEFIYKVKLRGTNF
PSDGPVMQKKTMGWEASSERMYPEDGALKGEIKQRLKLKDGGHYDAEVKTTYKAKKPVQLPGAYNVN
IKLDITSHNEDYTIVEQYERAEGRHSTGGMDELYKGGSASNDYTQQATQSYGAYPTQPGQGYSQQSS
QPYGQQSYSGYSQSTDTSGYGQSSYSSYGQSQNTGYGTQSTPQGYGSTGGYGSSQSSQSSYGQQSSY
PGYGQQPAPSSTSGSYGSSSQSSSYGQPQSGSYSQQPSYGGQQQSYGQQQSYNPPQGYGQQNQYNSS
SGGGGGGGGGGNYGQDQSSMSSGGGSGGGYGNQDQSGGGGSGGYGQQDRGGSGGTGGSGGTGGSGG
TADKQENGIKAEFTVRHNVEDGSVQLADHYQQNTPIGDGPVLLPDDHYLSTETVLSKDPNEKRDHMV
LHEYVNAAGITSKKGEELFTGVVPILVELDGDVNGHEFSVRGEGEGDATIGELTLKFICTTGELPVP
WPTLVTTLTYGVQCFSRYPDHMKRHDFFKSAMPEGYVQERTISFKDDGKYKTRAVVKFEGDTLVNRI
ELKGTDFKEDGNILGHKLEYNFNSHDVYIT

(Red: mCherry, Underline: FUS, Green: Homo-FC, Purple: flexible linker)

mCherry-WT VVD×2- 1.0 FUS

Wild type Vivid (WT VVD; amino acids 37-186)

HTLYAPGGYDIMGYLIQIMNRPNPQVELGPVDTSCALILCDLKQKDTPIVYASEAFLYMTGYSNAEV
LGRNCRFLQSPDGMVKPKSTRKYVDSNTINTMRKAIDRNAEVQVEVVNFKKNGQRFVNFLTMIPVRD
ETGEYRYSMGFQCETE

WT-VVD×2: WT-VVD-GGSLE-WT-VVD (Underline: linker)

mCherry-(CRY2 WT, CRY2olig,)-LAF or 1.0 FUS

CRY2WT (CRY2PHR 1-498)

MKMDKKTIVWFRRDLRIEDNPALAAAAHEGSVFPVFIWCPEEEGQFYPGRASRWWMKQSLAHLSQSL
KALGSDLTLIKTHNTISAILDCIRVTGATKVVFNHLYDPVSLVRDHTVKEKLVERGISVQSYNGDLL
YEPWEIYCEKGKPFTSFNSYWKKCLDMSIESVMLPPPWRLMPITAAAEAIWACSIEELGLENEAEKP
SNALLTRAWSPGWSNADKLLNEFIEKQLIDYAKNSKKVVGNSTSLLSPYLHFGEISVRHVFQCARMK
QIIWARDKNSEGEESADLFLRGIGLREYSRYICFNFPFTHEQSLLSHLRFFPWDADVDKFKAWRQGR
TGYPLVDAGMRELWATGWMHNRIRVIVSSFAVKFLLLPWKWGMKYFWDTLLDADLECDILGWQYISG
SIPDGHELDRLDNPALQGAKYDPEGEYIRQWLPELARLPTEWIHHPWDAPLTVLKASGVELGTNYAK
PIVDIDTARELLAKAISRTREAQIMIGAA

CRY2olig (E490G, point mutation form of CRY2 WT)

MKMDKKTIVWFRRDLRIEDNPALAAAAHEGSVFPVFIWCPEEEGQFYPGRASRWWMKQSLAHLSQSL
KALGSDLTLIKTHNTISAILDCIRVTGATKVVFNHLYDPVSLVRDHTVKEKLVERGISVQSYNGDLL
YEPWEIYCEKGKPFTSFNSYWKKCLDMSIESVMLPPPWRLMPITAAAEAIWACSIEELGLENEAEKP
SNALLTRAWSPGWSNADKLLNEFIEKQLIDYAKNSKKVVGNSTSLLSPYLHFGEISVRHVFQCARMK
QIIWARDKNSEGEESADLFLRGIGLREYSRYICFNFPFTHEQSLLSHLRFFPWDADVDKFKAWRQGR
TGYPLVDAGMRELWATGWMHNRIRVIVSSFAVKFLLLPWKWGMKYFWDTLLDADLECDILGWQYISG
SIPDGHELDRLDNPALQGAKYDPEGEYIRQWLPELARLPTEWIHHPWDAPLTVLKASGVELGTNYAK
PIVDIDTARELLAKAISRTRGAQIMIGAA

(RED : E490G)

1.0 LAF
MESNQSNNGGSGNAALNRGGRYVPPHLRGGDGGAAAAASAGGDDRRGGAGGGGYRRGGGNSGGGGGG
GYDRGYNDNRDDRDNRGGSGGYGRDRNYEDRGYNGGGGGGGNRGYNNNRGGGGGGYNRQDRGDGGSS
NFSRGGYNNRDEGSDNRGSGRSYNNDRRDNGGDG

1.0 DDX
MGDEDWEAEINPHMSSYVPIFEKDRYSGENGDNFNRTPASSSEMDDGPSRRDHFMKSGFASGRNFGN
RDAGECNKRDNTSTMGGFGVGKSFGNRGFSNSRFEDGDSSGFWRESSNDCEDNPTRNRGFSKRGGYR
DGNNSEASGPYRRGGRGSFRGCRGGFGLGSPNNDLDPDECMQRTGGLFGSRRPVLSGTGNGDTSQSR
SGSGSERGGYKGLNEEVITGSGKNSWKSEAEGGES

TAF15
MSDSGSYGQSGGEQQSYSTYGNPGSQGYGQASQSYSGYGQTTDSSYGQNYSGYSSYGQSQ
SGYSQSYGGYENQKQSSYSQQPYNNQGQQQNMESSGSQGGRAPSYDQPDYGQQDSYDQQSGYDQHQG
SYDEQSNYDQQHDSYSQNQQSYHSQRENYSHHTQDDRRDVSRYGEDNRGYGGSQGGGRGRGGYDKDG
RGPMTGSSGGDRGG

TIA1
INPVQQQNQIGYPQPYGQWGQWYGNAQQIGQYMPNGWQVPAYGMYGQAWNQQGFNQTQSSAPWMGPN
YGVQPPQGQNGSMLPNQPSGYRVAGYETQ

Dronpa 145N
SVIKPDMKIKLRMEGAVNGHPFAIEGVGLGKPFEGKQSMDLKVKEGGPLPFAYDILTTVFCYGNRVF
AKYPENIVDYFKQSFPEGYSWERSMNYEDGGICNATNDITLDGDCYIYEIRFDGVNFPANGPVMQKR
TVKWEPSTE**N**LYVRDGVLKGDVNMALSLEGGGHYRCDFKTTYKAKKVVQLPDYHFVDHHIEIKSHDK
DYSNVNLHEHAEAHSELPRQAK

GFP (-15 superfolder GFP)
KGEELFTGVVPILVELDGDVNGHEFSVRGEGEGDATIGELTLKFICTTGELPVPWPTLVTTLTYGVQ
CFSRYPDHMKRHDFFKSAMPEGYVQERTISFKDDGKYKTRAVVKFEGDTLVNRIELKGTDFKEDGNI
LGHKLEYNFNSHDVYITADKQENGIKAEFTVRHNVEDGSVQLADHYQQNTPIGDGPVLLPDDHYLST
ETVLSKDPNEKRDHMVLHEYVNAAGIT

RA
FDASNFKDFSSIASASSSWQNQSGSTMIIQVDSFGNVSGQYVNRAQGTGCQNSPYPLTGRVNGTFIA
FSVGWNNSTENCNSATGWTGYAQVNGNNTEIVTSWNLAYEGGSGPAIEQGQDTFQYVPTTE

STA
DPSKDSKAQVSAAEAGITGTWYNQLGSTFIVTAGADGALTGTYESAVGNAESRYVLTGRYDSAPATD
GSGTALGWTVAWKNNYRNAHSATTWSGQYVGGAEARINTQWLLTSGTTEANAWKSTLVGHDTFTK
VKPSAASIDAAKKAGVNNGNPLDAVQQ
