# Supplementary material for: Client proximity enhancement inside cellular membrane-less compartments governed by client-compartment interactions
Source: Nat Commun. 2020 Nov 6;11:5642. doi: 10.1038/s41467-020-19476-4 (PMC7648067; doi:10.1038/s41467-020-19476-4)
Supplement: Supplementary file 3 — Reporting Summary [file 41467_2020_19476_MOESM3_ESM.pdf]

nature research

Corresponding author(s): Yongwon Jung

Last updated by author(s): Oct 16, 2020

Reporting Summary

Nature Research wishes to improve the reproducibility of the work that we publish. This form provides structure for consistency and transparency in reporting. For further information on Nature Research policies, see our Editorial Policies and the Editorial Policy Checklist.

Statistics

For all statistical analyses, confirm that the following items are present in the figure legend, table legend, main text, or Methods section.

n/a	Confirmed	
☐	☒	The exact sample size (*n*) for each experimental group/condition, given as a discrete number and unit of measurement
☐	☒	A statement on whether measurements were taken from distinct samples or whether the same sample was measured repeatedly
☐	☒	The statistical test(s) used AND whether they are one- or two-sided<br>*Only common tests should be described solely by name; describe more complex techniques in the Methods section.*
☐	☒	A description of all covariates tested
☐	☒	A description of any assumptions or corrections, such as tests of normality and adjustment for multiple comparisons
☐	☒	A full description of the statistical parameters including central tendency (e.g. means) or other basic estimates (e.g. regression coefficient) AND variation (e.g. standard deviation) or associated estimates of uncertainty (e.g. confidence intervals)
☒	☐	For null hypothesis testing, the test statistic (e.g. *F*, *t*, *r*) with confidence intervals, effect sizes, degrees of freedom and *P* value noted<br>*Give P values as exact values whenever suitable.*
☒	☐	For Bayesian analysis, information on the choice of priors and Markov chain Monte Carlo settings
☒	☐	For hierarchical and complex designs, identification of the appropriate level for tests and full reporting of outcomes
☒	☐	Estimates of effect sizes (e.g. Cohen's *d*, Pearson's *r*), indicating how they were calculated

Our web collection on statistics for biologists contains articles on many of the points above.

Software and code

Policy information about availability of computer code

Data collection	ZEN 2.5 (Carl Zeiss) software were used for mammalian cell imaging and FRAP experiments.<br>Image J 1.52a(NIH) was used to select droplets above a certain size (Puncta size : > 0.01 µm2).
Data analysis	Commercially available software (Microsoft Excel 2016 and Origin 2018 (Originlab)) were used for statistical analysis.<br>Image J 1.52a(NIH) and ZEN 2.5 Lite (Carl Zeiss) were used for analyzing fluorescence intensity of mammalian cell imaging data.

For manuscripts utilizing custom algorithms or software that are central to the research but not yet described in published literature, software must be made available to editors and reviewers. We strongly encourage code deposition in a community repository (e.g. GitHub). See the Nature Research guidelines for submitting code & software for further information.

Data

Policy information about availability of data

All manuscripts must include a data availability statement. This statement should provide the following information, where applicable:

- Accession codes, unique identifiers, or web links for publicly available datasets
- A list of figures that have associated raw data
- A description of any restrictions on data availability

Source data for all figures are provided as a Source Data file.

Field-specific reporting

Please select the one below that is the best fit for your research. If you are not sure, read the appropriate sections before making your selection.

☒ Life sciences ☐ Behavioural & social sciences ☐ Ecological, evolutionary & environmental sciences

For a reference copy of the document with all sections, see nature.com/documents/nr-reporting-summary-flat.pdf

Life sciences study design

All studies must disclose on these points even when the disclosure is negative.

Sample size	We used sufficient sample sizes (> 30) obtained from three independent experiments for statistical comparisons. The 'central limit theorem' states that the distribution of sample means approximates a normal distribution as the sample size gets larger. Sample sizes (= 30 or > 30) are considered sufficient for the 'central limit theorem' to hold. The used sample size for each experiments were noted on a Source Data file.
Data exclusions	Cells with more than 20 passages and non-transfected cells were excluded from the data analysis.
Replication	Experimental findings were successfully reproduced in at least three independent experiments.
Randomization	All experiments were conducted with randomly selected samples without any biases or orders.
Blinding	No blinding was conducted, because blinding would not provide any reliable datasets for our biochemical study.

Reporting for specific materials, systems and methods

We require information from authors about some types of materials, experimental systems and methods used in many studies. Here, indicate whether each material, system or method listed is relevant to your study. If you are not sure if a list item applies to your research, read the appropriate section before selecting a response.

Materials & experimental systems

n/a	Involved in the study	
☒	☐	Antibodies
☐	☒	Eukaryotic cell lines
☒	☐	Palaeontology and archaeology
☒	☐	Animals and other organisms
☒	☐	Human research participants
☒	☐	Clinical data
☒	☐	Dual use research of concern

Methods

n/a	Involved in the study	
☒	☐	ChIP-seq
☒	☐	Flow cytometry
☒	☐	MRI-based neuroimaging

Eukaryotic cell lines

Policy information about cell lines

Cell line source(s)	ATCC was the source of A549, HEK293T and HeLa cell lines.
Authentication	The cell line (A549, HEK293T and HeLa) was not authenticated.
Mycoplasma contamination	The cells were not tested routinely for mycoplasma contamination.
Commonly misidentified lines (See ICLAC register)	Cell line used in this paper (A549, HEK293T and HeLa) are not listed in the ICLAC database.

April 2020
